# Three deaf mice: mouse models for TECTA-based human hereditary deafness reveal domain-specific structural phenotypes in the tectorial membrane

**DOI:** 10.1093/hmg/ddt646

**Published:** 2013-12-20

**Authors:** P. Kevin Legan, Richard J. Goodyear, Matías Morín, Angeles Mencia, Hilary Pollard, Leticia Olavarrieta, Julia Korchagina, Silvia Modamio-Hoybjor, Fernando Mayo, Felipe Moreno, Miguel-Angel Moreno-Pelayo, Guy P. Richardson

**Affiliations:** 1 School of Life Sciences, University of Sussex, Falmer, BrightonBN1 9QG, UK; 2 Unidad de Genética Molecular, Ramón y Cajal Institute of Health Research (IRYCIS); 3 BiomedicalNetwork Research Centre on Rare Diseases (CIBERER), Madrid, Spain

## Abstract

Tecta is a modular, non-collagenous protein of the tectorial membrane (TM), an extracellular matrix of the cochlea essential for normal hearing. Missense mutations in Tecta cause dominant forms of non-syndromic deafness and a genotype–phenotype correlation has been reported in humans, with mutations in different Tecta domains causing mid- or high-frequency hearing impairments that are either stable or progressive. Three mutant mice were created as models for human Tecta mutations; the Tecta^L1820F,G1824D/+^ mouse for zona pellucida (ZP) domain mutations causing stable mid-frequency hearing loss in a Belgian family, the Tecta^C1837G/+^ mouse for a ZP-domain mutation underlying progressive mid-frequency hearing loss in a Spanish family and the Tecta^C1619S/+^ mouse for a zonadhesin-like (ZA) domain mutation responsible for progressive, high-frequency hearing loss in a French family. Mutations in the ZP and ZA domains generate distinctly different changes in the structure of the TM. Auditory brainstem response thresholds in the 8–40 kHz range are elevated by 30–40 dB in the ZP-domain mutants, whilst those in the ZA-domain mutant are elevated by 20–30 dB. The phenotypes are stable and no evidence has been found for a progressive deterioration in TM structure or auditory function. Despite elevated auditory thresholds, the Tecta mutant mice all exhibit an enhanced tendency to have audiogenic seizures in response to white noise stimuli at low sound pressure levels (≤84 dB SPL), revealing a previously unrecognised consequence of Tecta mutations. These results, together with those from previous studies, establish an allelic series for Tecta unequivocally demonstrating an association between genotype and phenotype.

## INTRODUCTION

**Figure 1.**
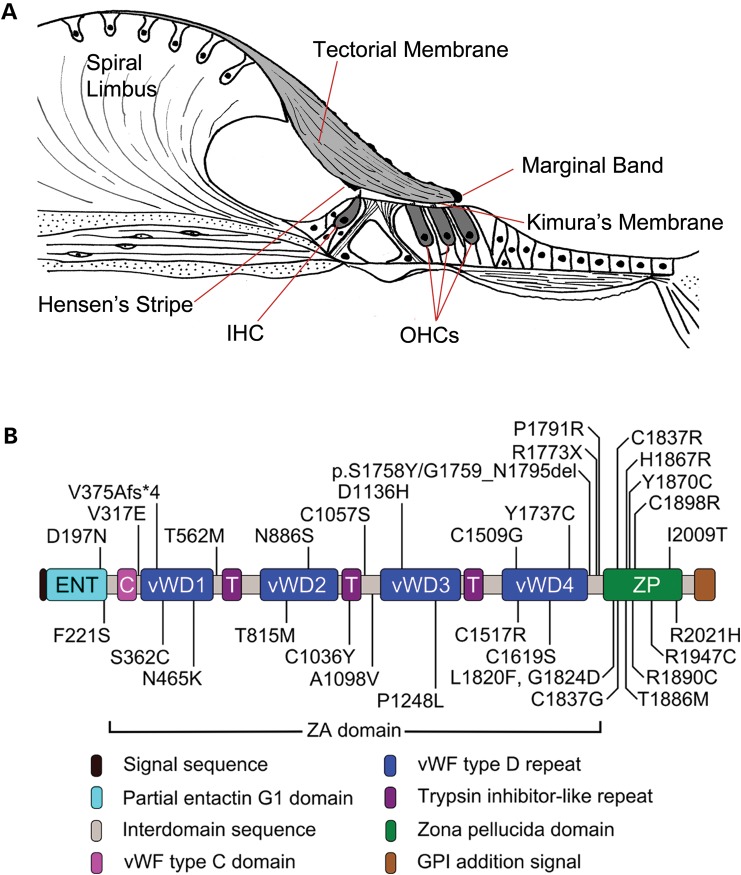
Structure of the organ of Corti and Tecta. (**A**) Schematic drawing depicting structure of the organ of Corti in the basal region of an adult mouse cochlea. The tectorial membrane attaches to the spiral limbus and, via Kimura's membrane, to the stereocilia of the OHC. Other features of the tectorial membrane include Hensen's stripe which lies medial to the inner hair cell (IHC) bundle and the marginal band which is situated at the lateral edge. (**B**) Domain structure of Tecta and location of deafness-causing missense mutations. Mutations in the entactin-G1 like domain, the vWFD1, vWFD2 and TIL2 repeats of the ZA domain and the ZP domain are associated with a mid-frequency hearing loss, while those in other regions of the ZA domain affect the high frequencies (20).

The tectorial membrane (TM) is a ribbon-like strip of extracellular matrix that extends along the entire length of the cochlea, attaching along its medial surface to the spiral limbus and laterally to the hair bundles of the sensori-motor outer hair cells (OHCs) in the organ of Corti (Fig. 1A). It is composed of three genetically distinct collagens, Types II, IX and XI, and five non-collagenous glycoproteins, α-tectorin (Tecta), β-tectorin (Tectb), otogelin, ceacam16, otogelinlike and otolin (1–7). The TM has been ascribed a number of distinct roles in hearing. These include acting as an inertial mass against which the hair cells can react, enabling the hair bundles of the OHCs to set their operating point, driving the hair bundles of the inner hair cells (IHCs), and increasing coupling along the length of the cochlea (8).

Autosomal dominant non-syndromic hearing loss (ADNSHL) has been mapped to 60 different loci and 24 of the genes involved have been identified thus far (http://hereditaryhearingloss.org, last accessed date on December 17, 2013). The causative gene at the DFNA8/12 locus is TECTA (NCBI Gene ID: 7007). The 13 individual missense mutations previously identified in TECTA (9–19) have been supplemented recently by an additional 20 novel missense mutations that were identified in a survey of Spanish and American families with ADNSHL. Mutations in TECTA (Fig. 1B) account for 4% of all ADNSHL cases and mutations at the DFNA8/12 locus are thought to be one of the major causes of ADNSHL (20).

Tecta is a large glycoprotein composed of multiple domains; an N-terminal entactin-G1-like domain, a central zonadhesin-like (ZA) domain comprising one von Willebrand factor type C repeat, four von Willebrand factor type D (vWF D) repeats and three trypsin inhibitor-like (TIL) repeats, and a C-terminal zona pellucida (ZP) domain (Fig. 1B) (20,21). Tectb is a much smaller glycoprotein consisting of a single ZP domain. Tecta and Tectb are both required for formation of the striated-sheet matrix (22,23), a laminated matrix within which the collagen fibrils of the TM are imbedded (24). ZP domain proteins are known to form filamentous structures such as the ZP of the mammalian oocyte (25), and ZA is a sperm receptor protein that binds to the ZP (26). These properties have led to the suggestion that Tecta and Tectb form filaments (either hetero- or homomeric) through their ZP domains that are cross-bridged by the ZA domains of Tecta to form the striated-sheet matrix (2). A more recent study has suggested that Ceacam16 mediates the interaction of Tecta/Tectb heteropolymers via the entactin-G1-like domain of Tecta (27).

The missense mutations identified in human Tecta are spread across every domain of the protein and produce different audiological phenotypes, allowing correlations between genotype and phenotype to be drawn. Hearing loss at the DFNA8/12 locus can be either pre or post-lingual in onset, stable or progressive, affect hearing in the mid or high-frequency ranges, and can vary from being mild to moderately severe (9). Correlations initially suggested mutations in the ZA domain affected high-frequency hearing, those in the ZP domain impaired mid-frequency hearing and mutations involving cysteine residues caused progressive hearing loss (9,10). The work of Hildebrand *et al.* (20) has refined this picture. Mutations in the entactin-G1-like domain, in the first two vWF D repeats and TIL2 repeat of the ZA domain and in the ZP domain are all associated with mid-frequency hearing loss, whilst mutations in other regions of the ZA domain result in high-frequency hearing loss. Mutations involving cysteine residues do not always cause progressive hearing loss.

Two mouse models for human DFNA8/12 mutations have been described thus far (21,28). In the Tecta^Y1870C/+^ mouse, a mouse with a missense mutation in the ZP domain of Tecta that is responsible for a moderate-to-severe (60–80 dB), prelingual, stable hearing deficit in an Austrian family, the TM remains associated with the organ of Corti but has a number of structural abnormalities (21). The neural masker tuning curves in the Tecta^Y1870C/+^ mice have a notch of insensitivity at the characteristic frequency of the probe tone that is similar in magnitude (60–70 dB) to the hearing loss reported in the affected Austrian family. In the Tecta^C1509G/+^ mouse, a mouse with a missense mutation in the ZA domains of Tecta that causes a mild–moderate (20–60 dB), progressive form of deafness in a Turkish family, the structural phenotype is more subtle and the auditory brainstem response (ABR) thresholds are raised by 25–40 dB across the hearing range, with the greatest loss being in the mid (10–35 kHz) frequency range (28).

Whilst these two mouse models have revealed the causes of deafness in the affected families, the extent to which the observed phenotypes are representative of those that might be expected from other deafness-causing missense mutations in the ZA and the ZP domains remains to be discovered. In this study we generated three additional Tecta mutant mice as models for clinically different DFNA8/12 phenotypes; Tecta^L1820F,G1824D/+^ as a model for ZP-domain mutations causing stable, mild-to-moderate, mid-frequency hearing loss in a Belgian family (11), Tecta^C1837G/+^ as a model for a ZP-domain mutation underlying progressive mid-frequency hearing loss in a Spanish family (13) and Tecta^C1619S/+^ as a model for a ZA-domain mutation responsible for progressive high-frequency hearing loss in a French family (12). Analysis shows that mutations in the ZP and the ZA domains cause distinct morphological phenotypes in the TM with a number of defects and differing degrees of hearing loss. The phenotypes of all three mutants are, however, stable for at least 6 months.

## RESULTS

**Figure 2.**
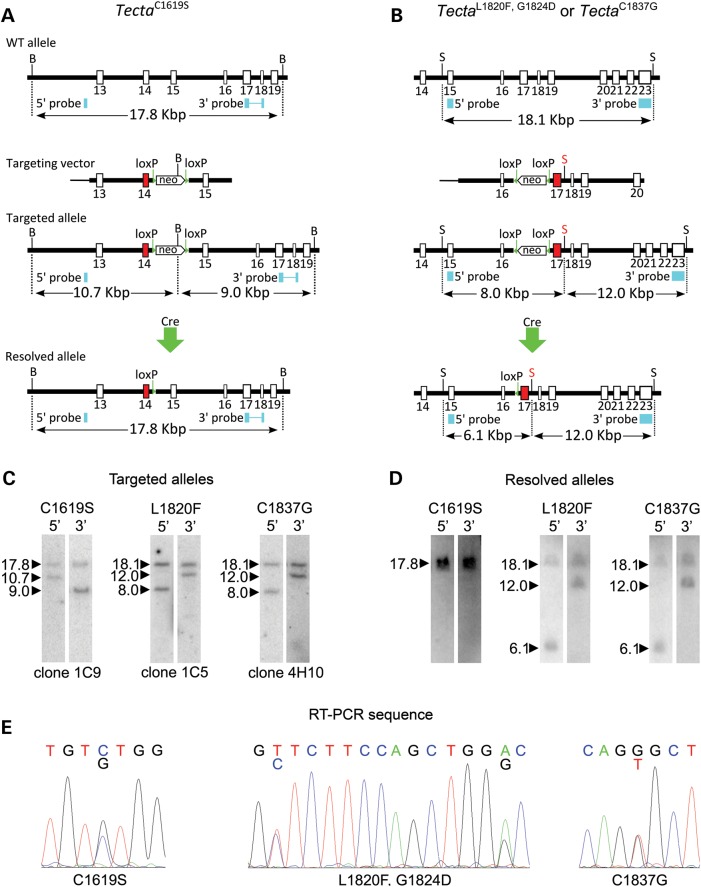
Generation of *Tecta* mutants by homologous recombination. Targeting vectors carrying point mutations in the ZA (**A**) or ZP domains (**B**) were composed of left and right arms flanking a floxed neo^R^ cassette. (**C**) Targeted ES cell lines were identified by Southern blotting. (**D**) Founder mice generated by blastocyst injection were mated with a Cre expressing mouse line to delete the selection cassette, leaving the point mutation and a single loxP site. (**E**) Direct sequencing of RT-PCR products was used to demonstrate the presence of the desired mutations in transcribed RNA. B, *Bam*HI; S, *Sac*I; loxP, loxP site; In **A** and **B** positions of 5′ and 3′ probes are marked with blue boxes, and the band sizes, in Kbp, identified by the probes on Southern blots are shown. Mutated exons are shown in red, exon 14 for Tecta^C1619S^, and exon 17 for Tecta^L1820F,G1824D^ and Tecta^C1837G^. The *Sac*I site between exons 17 and 18 in the targeting construct in **B** was introduced with a single base mutation to aid screening for recombinants.

Three mouse lines were made carrying point mutations in *Tecta* according to the strategies outlined in Figure 2A and B. Linearised targeting constructs were electroporated into mouse ES cells and Southern blotting was used to screen for correctly targeted recombinant clones. Individual ES clones were grown up and correct targeting confirmed by Southern blotting (Fig. 2C). Chimeric males derived by injecting ES cells into blastocysts were used to establish mouse lines containing the desired point mutations as well as the floxed Neo^R^ cassette used for ES cell selection. This selection cassette was removed by mating males with Cre deleter females to establish three lines carrying the desired point mutations and one loxP site in the flanking intron. Correct deletion of the Neo^R^ cassette was confirmed by Southern blotting (Fig. 2D) and PCR (not shown). The presence of the point mutations in Tecta transcripts was confirmed by direct sequencing of RT-PCR products (Fig. 2E). These transgenic mouse lines have the official designations Tecta^tm3.1Gpr^, Tecta^tm4.1Gpr^ and Tecta^tm5.1Gpr^ but are referred to throughout this paper by the point mutations introduced; thus Tecta^tm3.1Gpr^ is Tecta^C1619S^, Tecta^tm4.1Gpr^ is Tecta^L1820F,G1824D^ and Tecta^tm5.1Gpr^ is Tecta^C1837G^.

### Tectorial membranes of ZA- and ZP-domain mutants have distinct profiles that are stable with time

**Figure 3.**
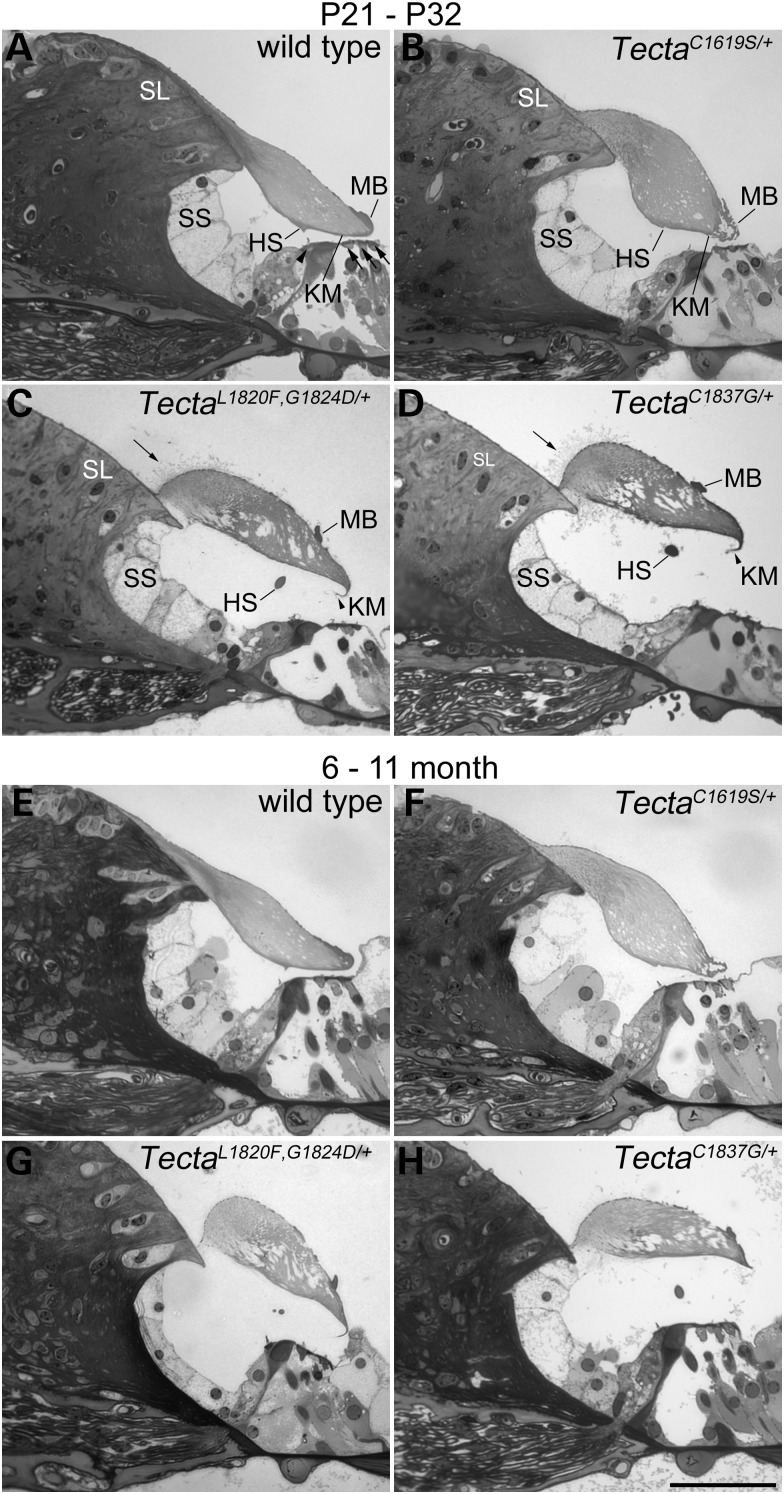
Structure of the organ of Corti in *Tecta* mutants. Toluidine blue stained sections from the basal coil (∼35 kHz region) of the organ of Corti of wild type and Tecta mutants at early (**A–D**, P21–P32) and late (**E–H**, 6–11 months) ages. (A P21, E 11 months) Wild type shows the characteristic tectorial membrane profile with attachment to spiral limbus (SL) and extension over the spiral sulcus (SS) to the hair cells (arrowhead points to IHC, arrows point to OHCs). A prominent Hensen's stripe (HS) is seen in close proximity to the IHC bundle and a marginal band (MB) covers the lateral edge of the tectorial membrane. (B P23, F 11 months) Tecta^C1619S/+^ mutant showing distorted cross-sectional profile of the tectorial membrane, with reduced limbal attachment, less distinct Hensen's stripe and fragmented marginal band. (C P21, G 6 months) Tecta^L1820F,G1824D/+^ and (D P32, H 8 months) Tecta^C1837G/+^. Both ZP domain mutants display a tectorial membrane with a ‘hump-backed’ cross-sectional profile, much reduced limbal attachment, a displaced marginal band and detached, malformed Hensen's stripe. In addition, Kimura's membrane (KM) appears delaminated and can sometimes be observed as a hook-like extension that curves back along the underside of the tectorial membrane (arrowheads). Collagen fibrils protrude from the upper surface in a region close to the lateral edge of the spiral limbus (arrows). There is not an obvious deterioration in structure as a function of age in any of the three mutants. Scale bar in H = 50 μm and applies to all panels.

**Figure 4.**
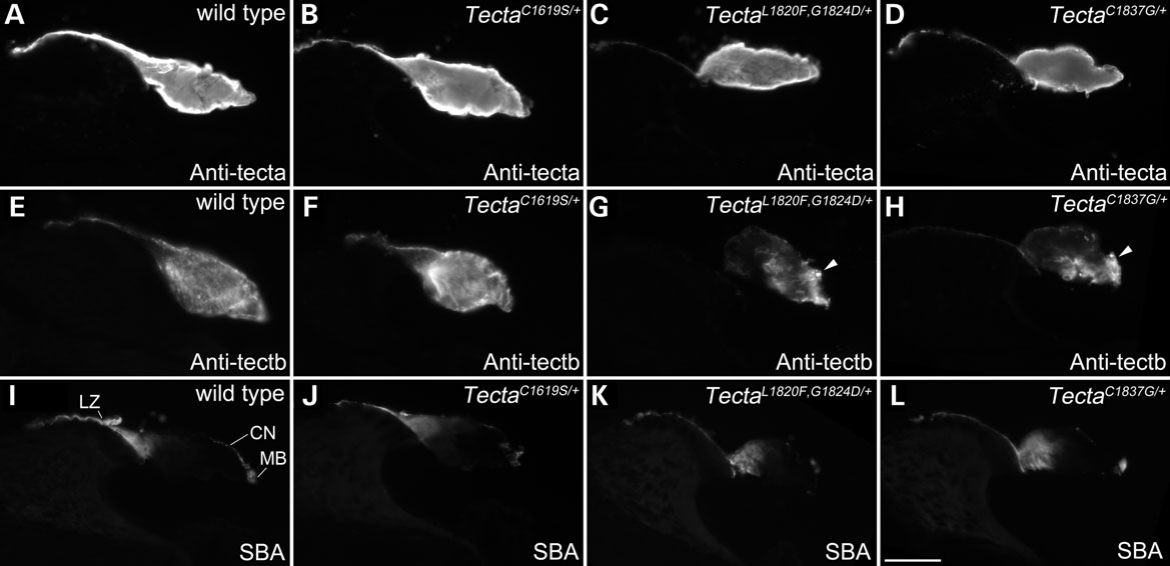
Distribution of tectorins and glycoconjugates in the TMs of *Tecta* mutants. Immunostaining with antibodies to Tecta (**A**–**D**) and Tectb (**E**–**H**) and lectin staining with SBA (**I**–**L**) on cryosections from the cochleae of 3-month-old wild type (A, E, I), Tecta^C1619S/+^ (B, F, J), Tecta^L1820F,G1824D/+^ (C, G, K) and Tecta^C1837G/+^ (D, H, L) mice. Images were obtained with a wide-field epifluorescence microscope. LZ = limbal zone, CN = covernet, MB = marginal band, indicated in **I**. Scale bar in **L**= 50 μm and applies to all panels.

**Figure 5.**
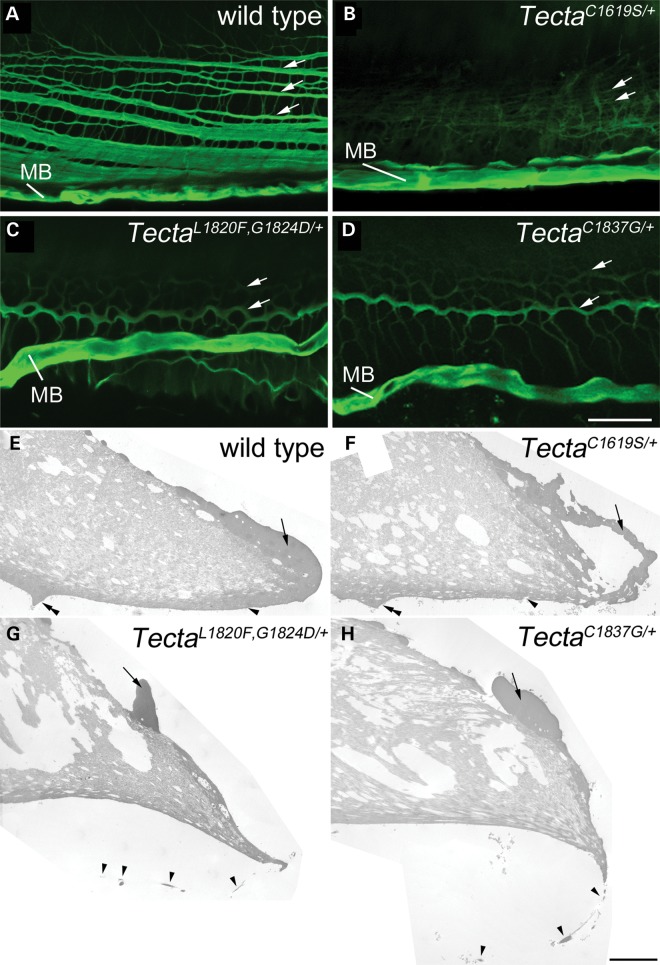
Covernet and marginal band in *Tecta* mutants. (**A–D**) Confocal *Z*-projections of SBA-labelled 60 μm thick horizontal sections of cochleae showing the pattern of covernet fibrils (longitudinal fibrils indicated by arrows) and marginal band (MB) in the tectorial membranes of wild type (A), Tecta^C1619S/+^ (B), Tecta^L1820F,G1824D/+^ (C) and Tecta^C1837G/+^ (D) adult mice. (**E–H**) TEM montages showing detail of the lateral region of basal tectorial membranes from wild type (E), Tecta^C1619S/+^ (F) Tecta^L1820F,G1824D/+^ (G) and Tecta^C1837G/+^ (H) mice. Arrow in each panel points to marginal band material, which is hollow in Tecta^C1619S/+^ mice (F) and is displaced in Tecta^L1820F,G1824D/+^ (G) and Tecta^C1837G/+^ (H) mice. Arrowhead in E points to Kimura's membrane, in F points to a hole in Kimura's membrane. In G and H arrowheads point to a delaminated region of Kimura's membrane. Double arrowheads point to Hensen's stripe in wild type (E) and Tecta^C1619S/+^ (F) mice. Hensen's stripe is dissociated from lower side of the tectorial membrane in Tecta^L1820F,G1824D^ (G) and Tecta^C1837G/+^ (H) mice and is out of the field of view. Scale bar in D = 20 μm and applies to A–D, scale bar in **H**= 5 μm and applies to E–H.

**Figure 6.**
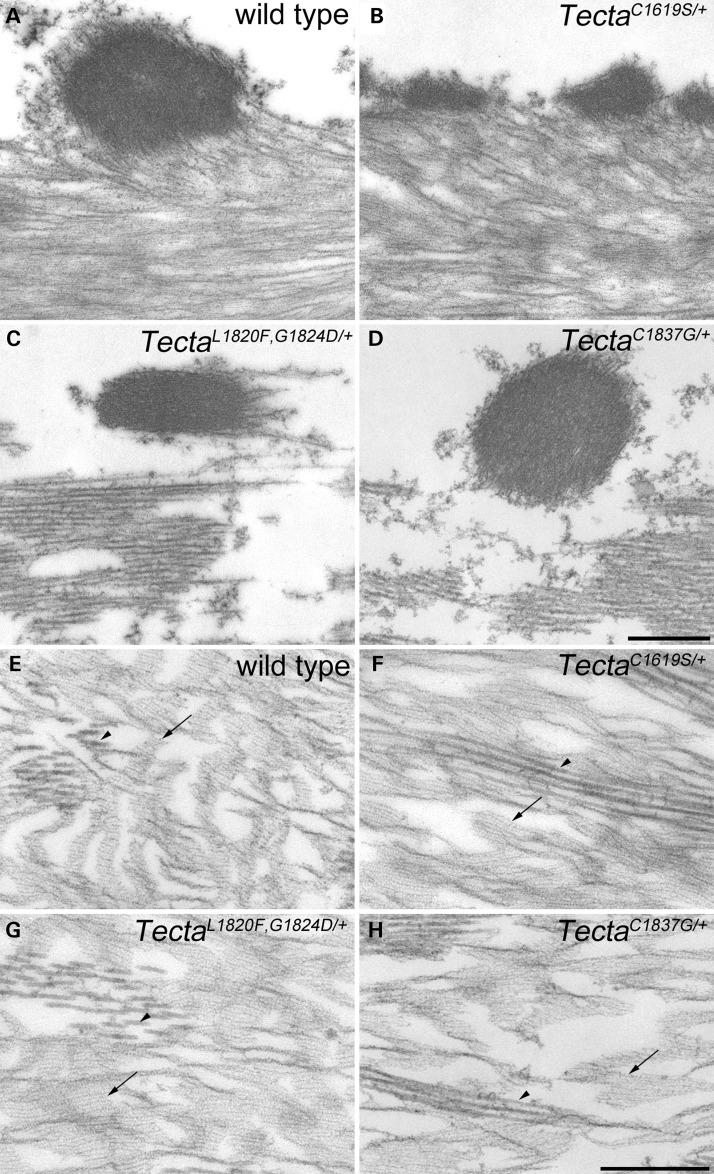
Covernet fibrils and striated-sheet matrix in *Tecta* mutants. (**A–D**) Transmission electron micrographs depicting covernet fibrils from basal tectorial membrane profiles in wild type (A), Tecta^C1619S/+^ (B), Tecta^L1820F,G1824D/+^ (C) and Tecta^C1837G/+^ (D) mice. In mice of all genotypes, covernet fibrils appear to be comprised of electron dense, compacted striated-sheet-like material. However, in Tecta^C1619S/+^ mice (B), the fibrils are more numerous and are of much smaller diameter than those of wild-type mice (A), whilst those of Tecta^L1820F,G1824D/+^ (C) and Tecta^C1837G/+^ (D) mice, although of normal cross-sectional appearance, are often partly separated from the underlying collagenous matrix. (**E–H**) Transmission electron micrographs depicting striated-sheet matrix (arrows) and collagen fibril bundles (arrowheads) from apical-coil tectorial membrane profiles in wild type (A), Tecta^C1619S/+^ (B), Tecta^L1820F,G1824D/+^ (C) and Tecta^C1837G/+^ (D) mice. In all genotypes striated-sheet matrix with a normal appearance is present in lateral regions of the tectorial membrane. Scale bar in D = 500 nm and applies to A–D, scale bar in H = 500 nm and applies to E–H.

Light, fluorescence and transmission electron microscopy (Figs 3–6) were used to analyse the structure of the TM and the organ of Corti in wild-type mice and in mice heterozygous for the three different missense mutations in Tecta. The morphology of the organ of Corti at early (P21–32, Fig. 3A–D) and later stages of life (6–11 months, Fig. 3E–H) is shown in Figure 3. A comparison of the two sets of micrographs indicates there is no change in the overall structure of the TM with time in either the wild type (Fig. 3A and E) or mutant (Fig. 3B–D, F–H) mice. In wild-type animals (Fig. 3A and E), the TM is attached via its medial, wedge-shaped limbal zone to the surface of the spiral limbus, stretches across the spiral sulcus, lies over the hair bundles of the IHCs and OHCs, and has a dense marginal band running along its lateral edge. In the basal, high-frequency end of the cochlea, a prominent, dense-staining ridge known as Hensen's stripe runs along the lower surface of the tectorial membrane in proximity to the hair bundles of the IHCs. In the Tecta^C1619S/+^ mouse (Fig. 3B and F), the cross-sectional profile of the TM is distorted, the limbal zone does not appear to extend fully across the surface of the spiral limbus in the medial direction, the marginal band is disrupted, and Hensen's stripe is not as prominent as it is in the wild-type mouse. In the Tecta^L1820F,G1824D/+^ and Tecta^C1837G/+^ mutant mice (Fig. 3C, D, G and H), the limbal zone is considerably reduced in its extent, the TM has a distinctive ‘hump-backed’ shape and the marginal band is displaced medially from the extreme lateral edge being located, instead, on the upper surface. Collagen fibrils project out of the medial region of the tectorial membrane, predominantly through the upper surface but also through the lower surface, just lateral to the spiral limbus. Additionally, Kimura's membrane, the dense lower surface of the TM, is delaminated and Hensen's stripe is no longer attached directly to the tectorial membrane (Fig. 3C, D, G and H). In the apical, low-frequency region of the cochlea in the Tecta^L1820F,G1824D/+^ and the Tecta^C1837G/+^ mice, the most apparent changes are in the cross-sectional profile and extent of limbal attachment (not shown). Apart from these changes in the form of the TM, the cellular organisation of the organ of Corti appears normal. The IHCs and OHCs are visible, and the tunnel of Corti presents with its normal A-frame shape provided by the twin pillar cells (Fig. 3A–H).

Fluorescence microscopy of cochlear cryosections was used to study the distribution of Tecta, Tectb and the glycoconjugates recognised by soybean agglutinin (SBA) in the TM (Fig. 4). Tecta is distributed throughout the core of the TM in the wild-type mouse and in all three mutant mice (Fig. 4A–D). Tectb is observed throughout the TM of both the wild type (Fig. 4E) and Tecta^C1619S/+^ (Fig. 4F) mouse, but is present at a lower level in the medial sulcal region of the TM in the Tecta^L1820F,G1824D/+^ (Fig. 4G) and the Tecta^C1837G/+^ (Fig. 4H) mouse. SBA stains the limbal zone and the marginal band of the TM in wild-type animals and all three mutants intensely (Fig. 4I–L). The covernet, a system of fibrils that run predominantly longitudinally along the length of the upper surface, is also stained by SBA in all genotypes but is more apparent in the wild-type mouse (Fig. 4I–L).

Confocal imaging of thick (60 µm) horizontal sections of the cochlea stained with SBA (Fig. 5A–D) and electron microscopy (Fig. 5E–H; Fig. 6A–D) reveal how all three mutations affect the covernet and the marginal band. In wild-type animals, the covernet fibrils aggregate laterally with one another and merge with the marginal band (Fig. 5A). The covernet is hard to discern in the Tecta^C1619S/+^ mouse (Fig. 5B), whilst in Tecta^L1820F,G1824D/+^ and Tecta^C1837G/+^ mice (Fig. 5C and D) the fibrils are reduced in number, more convoluted and run both longitudinally and radially across the surface of the TM, forming a mesh-like pattern. In both the Tecta^L1820F,G1824D/+^ and the Tecta^C1837G/+^ mouse, the marginal band of the TM is displaced medially and no longer runs along the lateral edge. Montages derived from low-magnification electron micrographs of the TMs of wild-type and Tecta mutant mice confirm that the marginal band remains closely associated with the main body of the TM in all three mutants despite being displaced medially in both the Tecta^L1820F,G1824D/+^ and Tecta^C1837G/+^ mice (Fig. 5E–H). Electron microscopy also reveals that Hensen's stripe lacks its typical pronounced V-shape in the Tecta^C1619S/+^ mouse (Fig. 5F), and confirms how Kimura's membrane is delaminated and fenestrated in both the Tecta^L1820F,G1824D/+^ and Tecta^C1837G/+^ mice whilst remaining attached to the far lateral edge of the tectorial membrane (Fig. 5G and H).

In wild-type mice, the covernet fibrils are continuous with the underlying striated-sheet matrix of the TM, and appear as electron-dense rods that can be a micron or more in diameter (Fig. 6A). In the Tecta^C1619S/+^ mouse, the fibrils are of much reduced diameter (Fig. 6B). By contrast, in the sulcal and more medial regions of the TM in the Tecta^L1820F,G1824D/+^ and Tecta^C1837G/+^ mice the covernet fibrils are still of large calibre but are less tightly associated with the main body of the TM (Fig. 6C and D). The central core of the wild-type TM is characterised by the presence of radially-oriented collagen fibrils embedded within a distinctive striated-sheet matrix structure (Fig. 6E). The striated-sheet matrix originates medially within the sulcal zone, extends to the marginal band and is particularly evident in apical regions of the cochlea, where it has a less compressed appearance. Although normal-looking striated-sheet matrix is present in the core of the TM in all three Tecta mutant mice (Fig. 6F–H), it is restricted to the lateral part of the TM in the Tecta^L1820F,G1824D/+^ and Tecta^C1837G/+^ mice (Fig. 6G and H) and absent from the sulcal region (Fig. 6C and D).

Within the limbal zone of the TM, the matrix surrounding the collagen fibrils is denser than the striated-sheet matrix that pervades through the core in more lateral regions. This dense matrix is present in the limbal zone of the Tecta^C1619S/+^ mouse, but is mostly missing from the severely reduced limbal zone of the TM in both the Tecta^L1820F,G1824D/+^ and the Tecta^C1837G/+^ mouse. Collagen fibrils erupt from the upper surface of the TM in the inner sulcal region in the Tecta^L1820F,G1824D/+^ and Tecta^C1837G/+^ mice, but remain embedded within the matrix of the TM in the Tecta^C1619S/+^ mouse (Supplementary Material, Fig. S2). In the Tecta^L1820F, G1824D/+^ and the Tecta^C1837G/+^ mice, collagen fibrils also occasionally extend out from the lower surface of the TM in the sulcal region immediately adjacent to the limbal attachment point (not shown).

An additional abnormality noted in the TM of the Tecta^C1619S/+^ mouse is the presence of exceptionally large diameter fibrils (Supplementary Material, Fig. S3) that have the characteristic collagen fibril banding pattern but can be 50 times thicker than the 20 nm collagen fibrils typically present in the TM. These fibrils are seen in the majority of Tecta^C1619S/+^ cochleae examined and whilst conspicuous are quite sparse in number (typically 1–5 per TM profile) and are often entirely absent from the basal half of the cochlea.

### Hair-cell numbers are stable in the mid-frequency region of Tecta mutants

**Figure 7.**
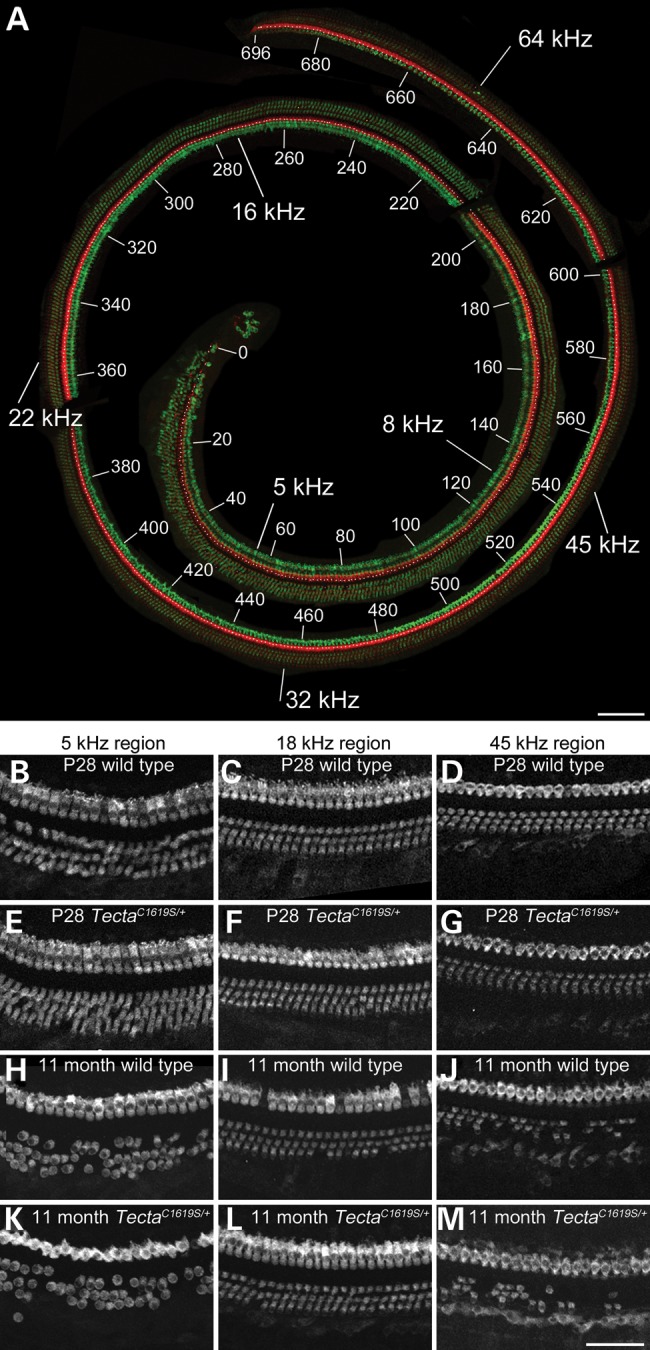
Hair-cell numbers in *Tecta* mutants. (**A**) An entire organ of Corti dissected from a decalcified P28 wild-type mouse cochlea by iterative trimming. Organ of Corti pieces were stained with phalloidin (red) and antibodies to Myosin VIIA (green) in order to visualise hair bundles and entire hair cells, respectively. The image was reconstructed from confocal stacks using Photoshop, and the organ of Corti trimmed out from the surround. Numbers 0–696 label IHCs, starting at the apical end. Values in kHz represent estimated best frequencies along the cochlea. (**B**–**M**) selected regions from the organ of Corti of P28 wild type (B, C, D), P28 Tecta^C1619S/+^ (E, F, G), 11-month-old wild type (H, I, J) and 11-month-old Tecta^C1619S/+^ (K, L, M) mice illustrating myosin VIIA staining. Little hair-cell loss is seen at P28 in any of the mice, whilst at 11 months, significant hair-cell loss is seen in extreme low-frequency and high-frequency (>45 kHz) regions of the cochlea in all mice. Scale bar in **A**= 100 μm, bar in **M**= 50 μm and applies to B–M.

The organs of Corti of Tecta^C1619S/+^ mice and those of wild-type littermates were dissected and hair-cell numbers were quantified using phalloidin and myosin VIIa staining at 1 and 11 months of age. Hair-cell numbers were also quantified for Tecta^L1820F,G1824D/+^ mice at 1 month, and for Tecta^C1837G/+^ mice and their wild-type littermates at 1, 8 and 13 months of age. Hair-cell loss was determined by gaps in the normal architecture and arrangements of hair and supporting cells in the organ of Corti, as seen with phalloidin staining, and was confirmed by an absence of myosin VIIa staining (Supplementary Material, Fig. S1). Quantitative analysis (Table 1) of whole-mount preparations of the organ of Corti (Fig. 7) revealed little hair-cell loss in any region of the cochlea at 1 month of age. At 8 months or older, sporadic hair-cell loss was observed in the apical-most region (encoding <12–14 kHz), and a near complete loss of hair cells was seen in the basal end of the cochleae (the region encoding greater than ∼55 kHz). Even at these older stages, little hair-cell loss was observed in the 20–45 kHz region. The amount of hair-cell loss was similar in both the wild-type and the heterozygous mutant mice at all ages.

**Table 1.** Hair-cell numbers in Tecta mutants

IHC region	21–120	121–220	221–320	321–420	421–520	521–620
OHC counts, per region in P30 wild-type cochleae						
Mean OHCs per region, *n* = 6	392 (±23)	347 (±12)	344 (±8)	326 (±5)	310 (±13)	322 (±11)
OHC loss per region, per genotype, juvenile						
P30 Wild type, *n* = 8	7.9 (±2.8)	3.1 (±1.5)	0.3 (±0.5)	0.3 (±0.5)	0.8 (±1.04)	0.5 (±0.9)
P30 Tecta^C1619S/+^, *n* = 5	7.8 (±2.2)	3.0 (±2.8)	0.0 (±0.0)	1.8 (±2.5)	0.6 (±0.6)	5.2 (±2.6)
P30 Tecta^L1820F,G1824D/+^, *n* = 4	14.3 (±3.3)	3.3 (±2.4)	0.3 (±0.5)	0.3 (±0.5)	1.0 (±0.8)	1.5 (±1.3)
P30 Tecta^C1837G/+^, *n* = 6	12.2 (±3.9)	4.7 (±4.5)	0.3 (±0.5)	0.8 (±1.6)	1.7 (±1.6)	0.5 (±1.2)
OHC loss per region, Tecta^C1837G^ mutation, 8-month adult						
8-month Wild type, *n* = 5	33.0 (±4.9)	20.0 (±5.9)	13.6 (±7.2)	5.2 (±3.0)	3.6 (±4.9)	*
8-month Tecta^C1837G/+^, *n* = 4	44.7 (±6.1)	9.3 (±1.5)	7.0 (±2.6)	7.3 (±1.5)	9.3 (±6.7)	*
OHC loss per region, Tecta^C1837G^ mutation, 13-month adult						
13-month Wild type, *n* = 4	56.8 (±15.0)	32.5 (±10.1)	19.3 (±8.4)	8.8 (±5/4)	11.0 (±5.0)	*
13-month Tecta^C1837G/+^, *n* = 2	60.0 (±5.7)	40.1 (±2.1)	19.5 (±3.5)	12.0 (±9.9)	9.5 (±0.7)	*
OHC loss per region, Tecta^C1619S^ mutation, 11-month adult						
11-month Wild type, *n* = 5	55.2 (±15.4)	44.8 (±16.1)	21.6 (±7.9)	9.0 (±4.6)	7.8 (±5.6)	*
11-month Tecta^C1619S/+^, *n* = 4	59.3 (±19.7)	28.3 (±18.6)	12.8 (±4.9)	23.0 (±15.3)	13.8 (±14.9)	*

Upper part shows the average number of OHCs in each region of 100 IHCs, starting at IHC 21 (numbered from the apical-most end), in P30 wild-type mice. The average number of missing OHCs in 1 month (P30) and 8–13-month-old wild type, Tecta^C1619S/+^, Tecta^L1820F,G1824D/+^ and Tecta^C1837G/+^ mice are shown below. Numbers in parentheses are standard deviations. *Values for region 521–620 in adult mice at 8 months or older were not determined due to the extensive amount of hair-cell loss (>50%) in this region for all genotypes.

### Reduced hair-bundle attachment in Tecta mutants

**Figure 8.**
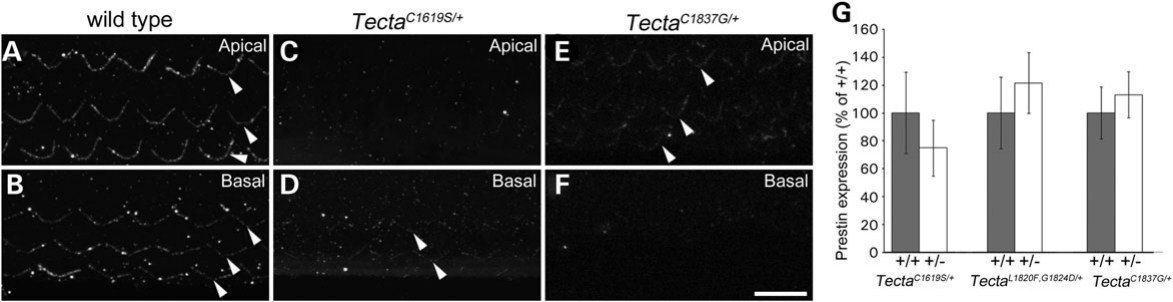
Hair-bundle imprints in TMs of *Tecta* mutants. Confocal Z-projections of stereocilin-labelled tectorial membranes from adult wild type (**A**, **B**), Tecta^C1619S/+^ (**C**, **D**) and Tecta^C1837G/+^ (**E**, **F**) mice showing location of individual rows of hair-bundle imprints (arrowheads). Three rows of hair-bundle imprints are visible in both the apical (A) and basal (B) coils of wild-type tectorial membranes. Imprints are not visible in the apical region of Tecta^C1619S/+^ tectorial membranes (C) whilst in basal regions (D) imprints are indistinct and are restricted to a maximum of two clear rows. In Tecta^C1837G/+^ tectorial membranes, imprints are visible but weakly stained in apical regions (E) and are not detected more basally (F). Scale bar = 10 μm and applies to all panels. (**G**) RT-qPCR measurement of prestin expression levels in wild type and Tecta^C1619S/+^, Tecta^L1820F,G1824D/+^ and Tecta^C1837G/+^ littermates. Mean prestin expression levels in heterozygous mutant mice are expressed as a percentage of the levels in wild-type littermates (*n* = 3 for each genotype). Error bars represent standard deviations.

In the Tecta^C1509G/+^ mouse, the TM is reduced in width and only contacts the first row of OHCs (28). To determine the extent to which the TM engages the OHCs in mice heterozygous for the three Tecta mutations, TMs were dissected from fixed, adult cochleae and stained with anti-stereocilin antibodies (29) in order to visualise the distribution of OHC-imprints on the lower surface of the TM (Fig. 8). In wild-type mice, three clear rows of tallest-row stereociliary imprints are consistently observed in both apical (Fig. 8A) and basal (Fig. 8B) regions of the TM. In the Tecta^C1619S/+^ mouse, imprints are not observed in apical, low-frequency regions and are only apparent in more basal regions, from the 30 kHz region and further basally (Fig. 8C and D). When observed, imprints in the Tecta^C1619S/+^ mouse are much more weakly stained than those of wild-type mice and generally consist of just two rows. Conversely, imprints in the TM of the Tecta^C1837G/+^ mouse (Fig. 8E and F) and the Tecta^L1820F,G1824D/+^ mouse (not shown) are only apparent in apical, lower-frequency regions, up to the 25–30 kHz area. As with the Tecta^C1619S/+^ mouse, these imprints are weakly labelled for stereocilin relative to those in wild-type controls.

### Prestin expression levels are unaffected in the Tecta mutant mice

In the Tecta^C1509G/+^ mouse, levels of the OHC motor protein prestin are elevated, possibly as compensation for the potential reduction in feedback caused by the decreased width of the TM (28). RT-qPCR was therefore used to determine levels of prestin mRNA in the three mutants and their wild-type littermate controls at P28. In all three mouse mutant lines prestin expression levels were similar to, and not significantly different from, those of wild-type littermates (Fig. 8G).

### Auditory thresholds are elevated but stable with time

**Figure 9.**
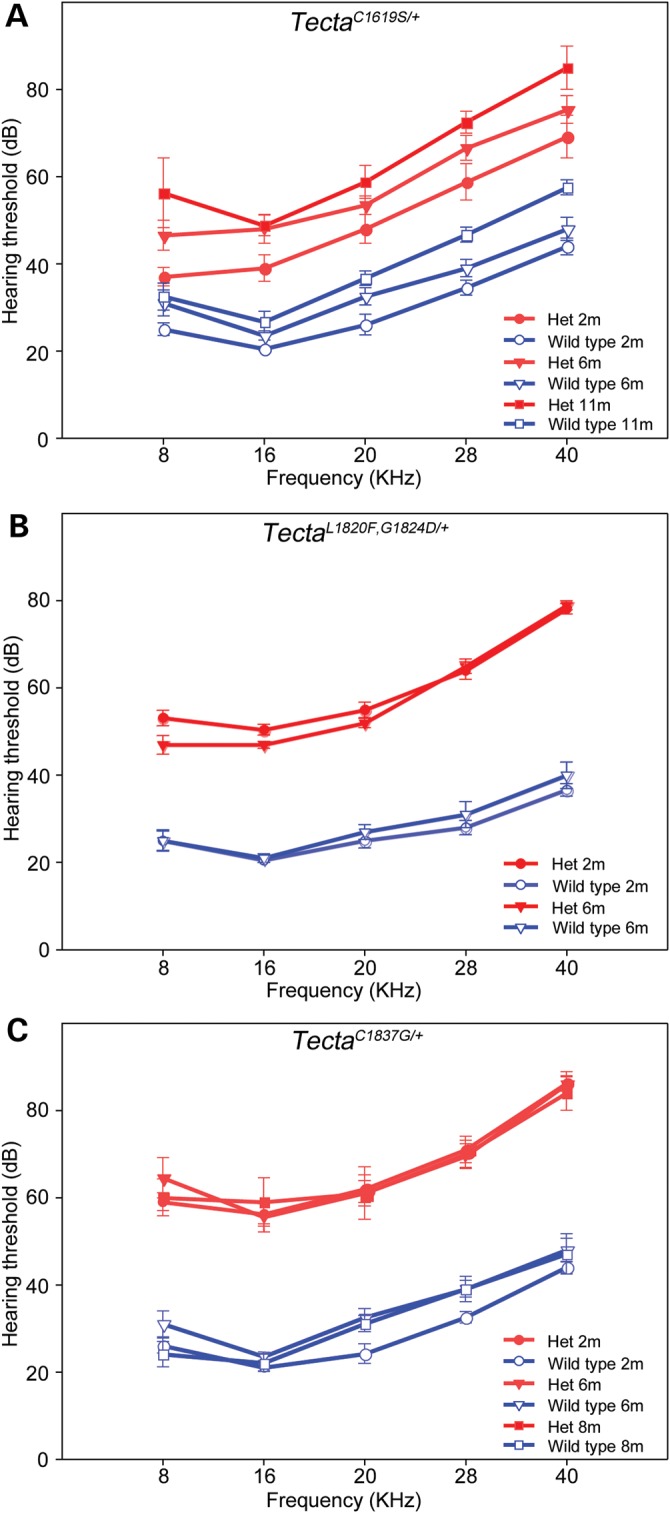
Auditory thresholds in *Tecta* mutants. ABR thresholds in T*ecta*^C1619S^, *Tecta*^L1820F,G1824D^ and *Tecta*^C1837G^ mutant mice were tested at 2 to 11 months of age. ABR thresholds in the 8-40 kHz range are elevated by 20–30 dB in *Tecta*^C1619S/+^ heterozygotes compared with wild-type controls, whilst those in the *Tecta*^L1820F,G1824D/+^ and *Tecta*^C1837G/+^ heterozygotes are elevated by 30–40 dB.

Auditory brain stem responses were used to assess the functional consequences of the three Tecta mutations. Measurements were made on mice from all three lines at 2–3 and 6 months of age. They were also made at 8 months of age for the Tecta^C1837G/+^ mouse, and at 11 months of age for the Tecta^C1619S/+^ mouse. Groups of heterozygous mutants and their wild-type littermates were tested for each mutant line, using different groups of animals for each time point. Responses were measured to a click stimulus, and to pure tones ranging in frequency from 8–40 kHz. In the wild-type S129SvEv mice ABR thresholds at 6 months of age were on average across all three lines 30 (8 kHz), 22 (16 kHz), 30 (20 kHz), 36 (28 kHz) and 44 (40 kHz) dB SPL. These values are within the limits reported for a large cohort of inbred mouse strains considered to have normal ABR thresholds (30), and would be consistent with this 129 strain being wild type at the *ahl/cdh23* locus (Legan, unpublished data). A small increase in ABR thresholds with time was observed in the wild-type animals from the Tecta^C1619S^ line (Fig. 9A), but not with the wild-type animals from either of the Tecta^L1820F,G1824D^ or the Tecta^C1837G^ lines (Fig. 9B and C). Heterozygotes from all three lines had ABR thresholds that were elevated uniformly across the frequency range, with the Tecta^C1619S/+^ mice showing the least severe loss and the Tecta^C1837G/+^ mouse showing the greatest. When averaged across all frequencies and all time points (see Table 2) there is hearing loss of 23.87 dB in the Tecta^C1619S/+^ mouse, 30.80 dB in the Tecta^L1820F,G1824D/+^ mouse and 35.10 dB in the Tecta^C1837G/+^ mouse. Hearing loss to a click stimulus shows the same trend (Table 2).

**Table 2.** Hearing loss in Tecta mutants

	Tecta^C1619S/+^	Tecta^L1820F,G1824D/+^	Tecta^C1837G/+^
Average hearing loss across all frequencies (±SD)			
2–3 months	20.2 ± 5.3 dB	32.8 ± 5.5 dB	37.3 ± 3.8 dB
6 months	25.8 ± 4.9 dB	28.8 ± 6.5 dB	32.6 ± 3.7 dB
8 months	–	–	35.4 ± 4.2 dB
11 months	25.6 ± 2.6 dB	–	–
Mean loss across all ages (±SD)	23.9 ± 3.2 dB	30.8 ± 2.8	35.1 ± 2.4 dB
Hearing loss to a click stimulus			
2–3 months	24.0 dB	50.0 dB	52.3 dB
6 months	29.0 dB	41.0 dB	48.0 dB
8 months	–	–	51.0 dB
11 months	33.0 dB	–	–
Mean loss across all ages (±SD)	28.7 ± 4.5 dB	45.5 ± 6.4 dB	50.4 ± 2.2 dB

### Tecta mutant mice show enhanced seizure sensitivity

**Figure 10.**
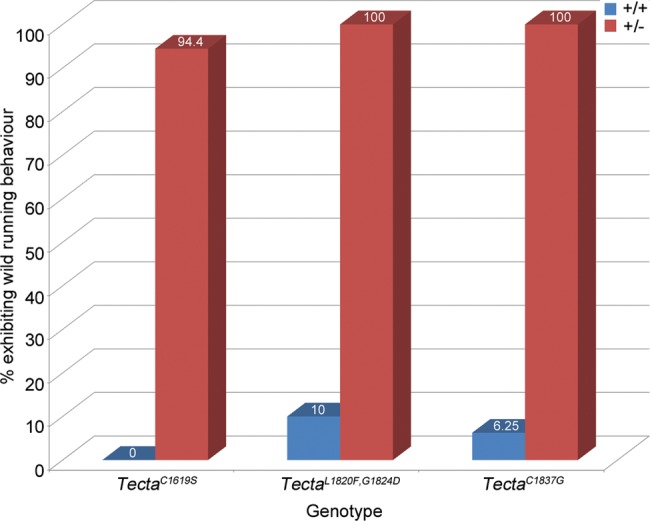
Seizure sensitivity of Tecta mutants. Percentage of wild-type and heterozygous Tecta mutant mice exhibiting wild running behaviour in response to 8–16 kHz white noise at a SPL ≤ 84 dB.

As ABR threshold shifts in mutants were stable with age, attempts were made to test whether interactions between environmental noise and the mutations were a cause of the progressive hearing loss reported in human subjects. Mice were therefore subjected to noise exposure paradigms reported to cause either temporary or permanent threshold shifts. In preliminary experiments, and in response to 8–16 kHz white noise at sound pressure levels (SPL) of 83 dB SPL or less, mice heterozygous for each of the three Tecta mutations unexpectedly exhibited wild running behaviour that was followed, within ∼10 s, by an audiogenic seizure. Tests on a small cohort of mice established that wild running behaviour was a reliable indicator of an impending audiogenic seizure. In order to reduce the severity of the procedure in all further experiments, the sound was turned off within ∼5 s of the onset of wild running behaviour. As shown in Figure 10, 94–100% of all heterozygous mice tested exhibited wild running behaviour predictive of a seizure with noise of ≤84 dB SPL. Of these heterozygotes, 84% (32/38) exhibited wild running with noise stimuli of ≤72 dB SPL. Only 0–10% of wild-type litter mate mice exhibited wild running with noise at or <84 dB SPL, with the rest being unaffected by noise stimulus levels of up to 102 dB SPL, the highest level tested.

### Homozygous phenotype

**Figure 11.**
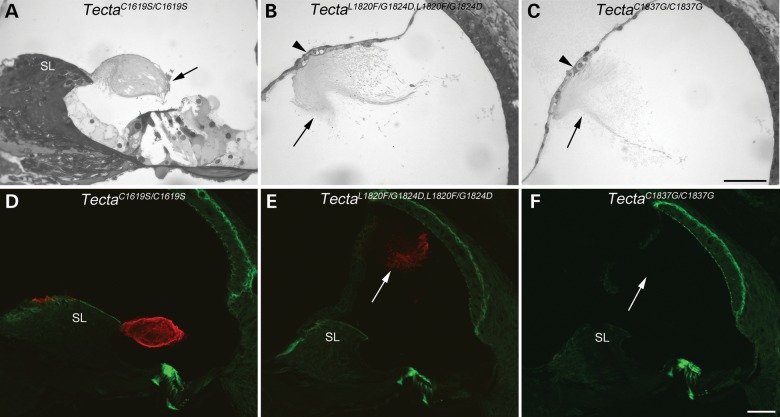
Structure of the organ of Corti in homozygous *Tecta* mutants. Toluidine blue stained resin sections (**A**–**C**) and anti-Tecta (red) and phalloidin (green) labelled cryosections (**D**–**F**) from basal cochlear coil of homozygous Tecta^C1619S/C1619S^ (A, D) Tecta^L1820F,G1824D/L1820F,G1824D^ (B, E) and Tecta^C1837G/C1837G^ (C, F) mutant mice. The tectorial membrane in the Tecta^C1619S/C1619S^ mouse (A, D) has reduced attachment to the spiral limbus (SL), a highly fragmented marginal band (arrow) but retains strong labelling for Tecta (D). The tectorial membranes of Tecta^L1820F,G1824D/L1820F,G1824D^ and Tecta^C1837G/C1837G^ mice are completely detached (arrows in B–C, E–F) from the spiral limbus and instead are associated with Reissner's membrane (arrowhead in B and C). Although the matrix of the tectorial membrane in the Tecta^L1820,G1824D/L1820F,G1824D^ mouse stains positively for Tecta (E) that in Tecta^C1837G/C1837G^ mutant does not (F). This may be reflected in the relative density of the tectorial membranes in the Toluidine blue sections (B, C). Scale bar in **C**= 50 μm and applies to A–C, scale bar in F = 50 μm and applies to D–F.

Light microscopy was used to examine the location and gross structure of the TM in mice homozygous for the three Tecta mutations (Fig. 11). In the Tecta^C1619S/C1619S^ TM mouse, the TM has a ‘hump-backed’ cross-sectional profile and a much reduced limbal attachment zone (Fig. 11A), a phenotype resembling that seen in mice heterozygous for the ZP domain mutations. The disruption of the marginal band seen in Tecta^C1619S/+^ mice is exacerbated in homozygous animals. TMs of both Tecta^L1820F,G1824D/L1820F,G1824D^ and Tecta^C1837G/C1837G^ animals are completely detached from the spiral limbus and are associated instead with Reissner's membrane (Fig. 11B and C). High levels of Tecta are detected by immunofluorescence in the TM of Tecta^C1619S/C1619S^ cochleae, and Tecta is also readily detectable in the detached TM of the Tecta^L1820F,G1824D/L1820F,G1824D^ mice (Fig. 11D and E). In the TM of the Tecta^C1837G/C1837G^ mouse, Tecta is virtually undetectable (Fig. 11F), although some weak labelling is seen at the extreme apical end (not shown). Similarly, Tectb staining is detected throughout the TM of the Tecta^C1619S/C1619S^ mouse, is present along the length of the TM of the Tecta^L1820F,G1824D/L1820F,G1824D^ mouse, but is restricted to the extreme apical end of the TM in the Tecta^C1837G/C1837G^ mouse (not shown).

## DISCUSSION

The phenotypic data from the mouse models for TECTA-based human hereditary deafness DFNA8/12 that have been generated thus far, and the clinical data from the corresponding human alleles, are summarised and compared in Table 3. A number of conclusions can be drawn from this information. First, a distinct genotype–phenotype correlation is emerging at the structural level. Second, at least two of the missense mutations involving cysteine residues reported to cause progressive hearing loss in humans do not cause a progressive, age-related deterioration in TM structure or an increase in ABR thresholds. Third, mice with missense mutations in Tecta are susceptible to audiogenic seizures.

**Table 3.** Phenotypic data for mouse and human Tecta alleles

			Tecta^C1509G/+^ (ZA)	Tecta^C1619S/+^ (ZA)	Tecta^L1820F,G1824D/+^ (ZP)	Tecta^C1837G/+^ (ZP)	Tecta^Y1870C/+^ (ZP)
Mouse	Structure of the organ of Corti	Tectorial membrane profile	Thicker and shorter	Distorted cross-sectional profile	‘Hump-backed’	‘Hump-backed’	‘Hump-backed’
		Limbal attachment	Reduced	Reduced	Much reduced	Much reduced	Much reduced
		Marginal band material	Dispersed and thinner	Hollow	Displaced	Displaced	Absent
		Hensen's stripe	ND	Less prominent	Malformed, detached	Malformed, detached	Absent
		Kimura's membrane	Fibrils loosely packed	Thinner, less distinct	Delaminated and hook-like	Delaminated and hook-like	Less firmly attached to TM
	Fluorescence microscopy	Tecta distribution	WT-like	Throughout the core of TM	Throughout the core of TM	Throughout the core of TM	Throughout the core of TM
		Tectb distribution	WT-like	WT-like	Reduced level in sulcal region	Reduced level in sulcal region	WT-like
	Transmission electron microscopy	Covernet fibrils	Covernet bundle is smaller	More numerous and smaller diameter	Partly separated from collagenous matrix	Partly separated from collagenous matrix	ND
	Hair cell	Hair-cell numbers	WT-like	WT-like	WT-like	WT-like	WT-like
		Hair-bundle attachment	1 Row	0 Rows of imprints in apical, 2 rows in basal	3 Rows of imprints in apical, 0 rows in basal	3 Rows of imprints in apical, 0 rows in basal	Imprints present, rows not evaluated
		Prestin level	Increased	WT-like	WT-like	WT-like	ND
	Auditory phenotype	ABR thresholds	Increased 25–40 dB	Increased ∼24 dB	Increased ∼30 dB	Increased ∼35 dB	ND
		Progression	ND	No	No	No	ND
		Seizure sensitivity	ND	Enhanced	Enhanced	Enhanced	ND
Human	Auditory phenotype	Auditory thresholds, severity	Increased, mild–moderate	Increased, mild–moderate	Increased, mild–severe	Increased, ND	Increased, moderate–severe
		Frequency range	High-frequency	High-frequency	Mid-frequency	Mid-frequency	Mid-frequency
		Progression	Yes	Yes	No	Yes	No

Table summarising the structural auditory phenotypes observed in mice carrying dominant, deafness-causing mutations in TECTA and the auditory phenotype of the corresponding human alleles. Data for the Tecta^C1619S^, Tecta^L1820F,G1824D^ and Tecta^C1837G^ mice are from the current study; data for the Tecta^C1509G^ mouse are from Xia *et al*. (28), and data for the Tecta^Y1870C^ mouse are from Legan *et al.* (21). WT = wild type, ND = not determined, TM = tectorial membrane. Data for human alleles are from refs (11 –13) and (17).

The structural defects observed in the TMs of the Tecta^L1820F,G1824D/+^ and Tecta^C1837G/+^ mice, both of which have mutations in the ZP domain, are very similar to those previously described for the Tecta^Y1870C/+^ ZP-domain mutant mouse (21). The common features include a much reduced limbal zone, the absence of striated-sheet matrix and a disruption of collagen fibril organisation in the sulcal region, a delamination of Kimura's membrane, a detachment of Hensen's stripe, and a change in the form of the covernet. Although the marginal band was previously reported to be absent in the Tecta^Y1870C/+^ mouse, rather than medially displaced along the upper surface as it is in the Tecta^L1820F,G1824D/+^ and the Tecta^C1837G/+^ mice, a retrospective analysis reveals that the marginal band is completely detached from the TM in the Tecta^Y1870C/+^ mouse and is to be found floating freely within scala media (Goodyear, unpublished data). The structural defects observed in the TM of the Tecta^C1619S/+^ mouse are quite different from those observed in the ZP-domain mutants, but similar in many respects to those described by Xia *et al.* (28) for the Tecta^C1509G/+^ mouse. The similarities include a disruption of the marginal band, a severe reduction in the covernet, a distortion of the cross-sectional profile and reduced attachment to OHCs. It can therefore be concluded, on the basis of the five mouse models for DFNA8/12 that have been created to date, that there is clear genotype–phenotype correlation, with ZA-domain mutations and ZP-domain mutations causing distinct structural phenotypes.

The ABR threshold measurements made in this study indicate the hearing loss (across all frequencies and ages tested) is, on average, slightly greater (∼10 dB) in the two ZP domain mutants than it is the ZA domain mutant. ABR thresholds in the ZA domain mutant described by Xia *et al.* (28) were elevated by 25–40 dB across the 10–40 kHz range, with hearing loss being greatest in the mid-frequency (16–22 kHz) range. By contrast, ABR thresholds in the Tecta^C1619S/+^ ZA mutant described in this study were uniformly elevated (by ∼24 dB) across the entire (8–40 kHz) range when tested at 11 months of age. Whilst ABR thresholds were not measured in a previous study (21) of the Tecta^Y1870C/+^ ZP domain mutant mouse, the comparable neural (compound action potential) thresholds were elevated by an average of 55 dB across the 5–30 kHz range, a loss higher than the 30–35 dB elevation in ABR thresholds found in this study with the Tecta^L1820F,G1824D/+^ and the Tecta^C1837G/+^ mice. Thus, whilst ZA and ZP domain mutations generate two distinct morphological phenotypes, there are subtle differences in severity within each group. Some of these subtleties may be attributed to degrees of morphological disruption. For example, the more severe hearing loss encountered in the Tecta^Y1870C/+^ mouse relative to that encountered in either the Tecta^L1820F,G1824D/+^ or the Tecta^C1837G/+^ mouse may be due to the complete detachment of the marginal band from the TM observed in the former. Also, the more severe hearing loss reported for the Tecta^C1509G/+^ mouse relative to that of the Tecta^C1619S/+^ mouse may be due to the number of rows of OHCs that contact the TM, one row in the Tecta^C1509G/+^ mouse versus two rows in the Tecta^C1619S/+^ mouse.

The observed correlation between the Tecta protein domain in which the missense mutations occur (referred to here as the genotype) and the morphological phenotype observed in heterozygotes suggests that the ZA domain is vital for the normal development of the marginal band, covernet and Hensen's stripe. In contrast, the ZP domain is more critical for ensuring these peripheral features are correctly placed or attached to the TM. Furthermore, mutations involving cysteine residues in the fourth vWF type D repeat of the ZA domain have little, if any, effect on the structure of the striated-sheet matrix, whilst mutations in the ZP domain only impinge upon the integrity of the striated-sheet matrix within the medial region of the TM, and not in the more lateral regions. The phenotype observed in mice homozygous for these missense mutations is different from that observed in heterozygotes and, for the ZP domain mutations, largely similar to that encountered in mice homozygous for a functional null allele of Tecta (the Tecta^ΔENT^ allele). The mutations are therefore semi-dominant. It seems unlikely, however, that the heterozygous phenotype can be accounted for by haplo-insufficiency as Tecta can be detected in the TMs of mice homozygous for these mutations, and mice that are heterozygous for either the Tecta^ΔENT^ allele (21) or true null alleles of Tecta (Legan, Goodyear and Richardson, unpublished data) have TMs that are very similar in appearance to those of wild-type mice.

Two of the mice created in this study, the Tecta^C1837G/+^ and the Tecta^C1619S/+^ mice, have missense mutations that involve the loss of cysteine residues and were generated as models for TECTA mutations that are reported to cause progressive, as opposed to stable, forms of human deafness. There was not, however, any evidence for a deterioration in TM structure with time in the mutant mice, nor was there an increase in the hearing thresholds over the periods during which ABR thresholds were monitored. Whilst a small sporadic loss of hair cells was noted in the 10–12 kHz region of the cochlea with age in wild-type mice and mice of all three genotypes, the frequency range over which the ABRs were measured (from 8–40 kHz) is, for the most part, encoded by a region of the cochlea within which there is no evidence for a loss of OHCs. Although the time periods studied (over 5 months for the Tecta^C1837G/+^ mouse and over 8 months for the Tecta^C1619S/+^ mouse) may be considered to be rather short, they do represent 20–33% of the life span of a typical laboratory mouse. Assuming ageing can be scaled proportionally, the 8 and 11-month-old mice would be equivalent to humans of ∼25 and ∼36 years of age (assuming life spans of 2 and 80 years for mice and humans, respectively), ages at which progression of the hearing loss has been detected in both the Spanish and the French families. Whilst it is possible that the lack of a progressive phenotype in these mice is simply due to the fact that they do not live long enough, there are certainly other possibilities. For example, other genetic traits may segregate and interact with the TECTA mutations in the human population to cause a progressive phenotype. Likewise it is possible that a progressive phenotype would be observed if the mice were to be crossed onto other genetic backgrounds.

The seizure sensitivity of the Tecta mice meant it was not possible to examine whether exposure to high noise levels would exacerbate the observed structural effects of the mutations. It was also an unexpected observation, considering the elevated auditory thresholds of the mutants and the distinct morphological phenotypes. There are several possible explanations for this observation that will be the focus of future studies. For example, a reduction in auditory input during the development of the auditory system in the mutants may lead to an increase in the innervation of some or all of the central auditory nuclei making the animals hypersensitive to acoustic stimuli, the octave band noise stimulus may set up an unusual resonance in the cochlea when the TM is malformed, or Tecta may be expressed in the brain. A number of array datasets in the GEO database provide evidence that Tecta is expressed at low levels in the mouse brain, and the Allen brain atlas indicates there may be expression in several locations in the brain (Tecta—RP_060315_04_H11). Close inspection of the high-resolution images in the Allen atlas reveals, however, that these signals are likely to be artefacts, and there is no evidence for Tecta expression in the brain in the EMAGE database (EMAGE: 17941). The level and location of Tecta expression in the brain is therefore open to debate. Although an explanation for the observed seizure sensitivity is thus far lacking, it should be noted that the Tecta mutant mice all carry mutations that are known to cause deafness in humans, and the affected patients might also be abnormally sensitive to certain sound stimuli. Whilst the effects of TECTA mutations are reported to be non-syndromic and only cause deafness, subtle sub-clinical phenotypes cannot be excluded.

In conclusion, the results of this study provide firm evidence that there is an association between the Tecta domain in which the missense mutation occurs and the resultant morphological defect, suggesting the different modules differ in their capacity for shaping the structure of the tectorial membrane, an extracellular matrix with a precisely regulated form and size critical for normal cochlear function.

## MATERIALS AND METHODS

All animal procedures at the University of Sussex were performed under UK Home Office Project licences (PPL70/6721 entitled ‘Molecular, Cellular and Physiological Basis of Hearing and Deafness’ and PPL70/7658 entitled ‘Molecular and Cellular Basis of Hearing and Deafness’) and with the approval of The University of Sussex Ethical Review Committee. At the Institute of Biomedical Research Alberto Sols and Centre for Biomedical Network Research on Rare Diseases (CIBERER) in Madrid, animals were housed following the recommendations of Federation of European Laboratory Animal Science Associations and animal experimentation was conducted in accord with Spanish and European legislation (EU directive 2010/63/EU) and approved by the Instituto Ramon y Cajal de Investigaciones Sanitarias (IRYCIS) Ethical Review Committee and by the Animal Care and Use Committees of Spanish National Research Council (CSIC).

### Introducing DFNA8/12 point mutations into mouse Tecta by homologous recombination in ES cells

Human (NM_05422_2) and mouse (NM_009347) Tecta mRNAs both encode proteins of 2155 amino acids that share 95.9% sequence identity. The open reading frames of both mRNAs align with no gaps, and consequently human TECTA mutations directly map onto mouse Tecta. Three Tecta mouse mutants were generated by homologous recombination in ES cells that correspond to the human mutations: c.4856G>C (p.C1619S); c.5458C>T, c.5471G>A (p.L1820F, p.G1824D); and c.5509T>G (p.C1837G). Three targeting vectors carrying point mutations were constructed in pBluescript by standard techniques and were composed of left and right arms flanking a floxed neomycin resistance cassette (Fig. 2A–B).

To make the c.4856G>C (p.C1619S) targeting vector, a 10.6 kb XhoI fragment of the mouse Tecta gene spanning exons 13–15 was isolated from the 251B121GPCH15 BAC and cloned into the pUC19 vector. A PmeI-SnaBI fragment was isolated and ligated into pBluescript II KS, the c.4856G>C mutation was introduced by site-directed mutagenesis at exon 14, and the mutagenized fragment was cloned back into the original pUC19 vector. A *Not*I linker was then introduced into the PmeI site of the final vector in which the loxP-neoR-loxP cassette flanked by NotI sites was inserted.

The c.5458C>T, c.5471G>A (p.L1820F, p.G1824D) and c.5509T>G (p.C1837G) targeting constructs were built from a 10.7 Kb *Aat*II to *Sac*II fragment of the mouse Tecta gene, spanning exons 16 to 20, that was isolated from the CITB CJ7 BAC clone 251B12 (129S1/Sv) and ligated into pUC18. A *Sma*I–*Sph*I fragment of 1.9 Kb was then isolated and cloned into pBluescript II KS. Site-directed mutagenesis was used to introduce either the c.5458C>T and c.5471G>A (p.L1820F, p.G1824D) mutations into exon 17 or the c.5509T>G (p.C1837G) mutation into exon 17 and the mutagenised fragments were then cloned back into the original vector. Then a NotI linker was introduced into a *Sma*I site enabling a loxP-neoR-loxP cassette flanked by NotI sites to be inserted into the correctly mutagenised vector.

Restriction mapping and DNA sequencing was used to confirm each step of vector construction was correctly carried out for all three vectors.

### Targeting mouse ES cells

Vectors were linearised with PmlI (c.4856G>C (p.C1619S)) or *Sac*II (c.5458C>T, c.5471G>A (p.L1820F, p.G1824D) and c.5509T>G (p.C1837G)) and electroporated into mouse ES cells (CCB line, S129SvEv) and resistant colonies were selected as described (Legan *et al*., 2005). Targeted ES cell lines were identified by Southern blotting using probes external to the vector homology arms. Probes for c.4856G>C (p.C1619S) were amplified by the PCR using primers E14probeA2F1 (GCCTTCTACCTCCCTGAGCAAGGAT) with E14probeA2R1 (TCTCAGCATCGGGTCACCTACTGCA) and E14probeB2F1 (GCGTAAGCCGATGCCTCTGTGTGTA) with E14probeB2R1 (CCTGGACTCACGTGGTTCTGACTGA). Probes for c.5458C>T, c.5471G>A (p.L1820F, p.G1824D) and c.5509T>G (p.C1837G) were amplified with primers 83GF3 (CATGTGACAATGTGCACATC) and MG492-1351R (GACGTCATGGTGGAGCTGAG), and maZPΔF1 (TTATGTGGCTGCATTTAACGAACTCAGGGT) and maΔZPR2 (ATCAGACTTCTGTAGCCAGA). PCR products were gel purified and random primer labelled (GE Healthcare, UK) with α^32^P-dCTP (Amersham, UK). Potential targeted clones were individually grown and rescreened by Southern blotting to confirm targeting.

Correctly targeted ES cell lines were injected into C57bl6J blastocysts, and transferred to pseudopregnant recipient mice. The resulting chimeric mice were tested for germ line transmission then mated to the beta actin Cre mouse to delete the selection cassette, leaving the point mutation and a single loxP site in the genome.

### Light and electron microscopy

Mice were killed with an overdose of anaesthetic and the labyrinths were rapidly removed from the head and placed in PBS. Following removal of the oval and round windows, and after making a small hole through the bone at the apical end of the cochlea, ∼20 µl of fixative (2.5% glutaraldehyde, 1% tannic acid in 0.1 m sodium cacodylate pH 7.2) was perfused gently through the oval window of each labyrinth, followed by a further 20 µl of fixative via the apical hole. The labyrinths were then immersed in fixative for 4 h at room temperature followed by ∼12 h at 4°C and, after three washes (5 min each) in 0.1 m sodium cacodylate buffer pH 7.2, were postfixed by immersion in 1% osmium tetroxide for 2–4 h at room temperature. Tubes containing the samples were continuously rotated during the fixation steps. Following osmication samples were washed with cacodylate buffer and then decalcified in 0.5 M EDTA pH 8.0 containing 0.25% glutaraldehyde at 4°C until the bony capsule was soft, ∼2–5 days depending upon the age of the tissue. Once decalcified, samples were washed briefly in H_2_O, dehydrated through a series of ascending concentrations of ethanol, equilibrated with propylene oxide, infiltrated with and finally imbedded in epoxy resin (TAAB 812 resin). Resin was cured at 60°C for 24 h, and blocks were sectioned using a Reichert Ultramicrotome. One micron thick sections were cut with glass knives, stained with 1% Toluidin Blue for light microscopy, viewed with a Zeiss Axioplan microscope, and the images were captured with a Spot RT slider digital camera. Ultrathin (∼90 nm thick) sections were cut with a diamond knife, mounted on copper mesh grids, double stained with uranyl acetate and lead citrate, and viewed with a Hitachi 7100 transmission electron microscope operating at 100 kV. Images were captured with a Gatan Ultrascan 1000 CCD camera. Where necessary, groups of images were photomontaged using the Photomerge feature of Adobe Photoshop CS5. Cochleae were examined from early maturation through to advanced age, *n* number for approximate age groups for light microscope analysis are as follows: wild-type 1 month (*24*), 2 months (*8*), 6–8 months (*12*), 11+ months (*10*); Tecta^C1619S/+^ 1 month (*6*), 2 months (*3*), 6–8 months (*4*), 11+ months (*6*), Tecta^L1820F,^^G1824D/+^ 1 month (*5*), 2 months (*3*), 6–8 months (*8*), Tecta^C1837G/+^ 1 month (*9*), 2 months (*3*), 6–8 months (*6*), 11+ months (*3*). For TEM analysis, total *n* numbers examined from a range of selected ages were: wildtype (*17*), Tecta^C1619S/+^ (*10*), Tecta^L1820F,G1824D/+^ (*7*) and Tecta^C1837G/+^ (*11*).

### Fluorescence microscopy of cryosections

Labyrinths were placed in PBS and, after removal of the oval and round windows, immersion fixed in 4% formaldehyde in 0.1 M sodium phosphate buffer pH 7.2 for 2 h at room temperature. Following 3 washes in PBS, samples were decalcified in 0.5 M EDTA for 2–5 days at 4°C, equilibrated in 30% sucrose in PBS overnight at 4°C, and imbedded in 1% low gelling temperature agarose (Sigma Type VII) dissolved in PBS containing 18% sucrose. Agarose blocks containing the labyrinths were frozen onto microtome chucks using Cryospray 134 (TAAB), and sectioned at 10–60 µm thickness at a temperature of −30°C in a cryostat. Cryosections were dried onto gelatine coated glass microscope slides at 37°C for 1–2 h prior to staining with lectins or antibodies. For lectin staining, sections were pre-blocked with 3% BSA in PBS; for antibody staining, sections were pre-blocked with 10% horse serum in PBS. Following 1–2 h in pre-block, sections were stained overnight at room temperature with primary antibodies or FITC conjugated lectins diluted in the appropriate pre-block. Primary antibodies were detected with FITC or Alexa-488 conjugated anti-rabbit antibodies diluted in pre-block containing horse serum. Texas Red conjugated phalloidin was added to the secondary anti-antibody or the lectin in order to visualise the distribution of F-actin. Images of fluorescent staining were captured with a Zeiss Axioplan widefield microscope equipped for epifluorescence microscopy or with a Zeiss LSM 510 confocal microscope. Antibodies used were: Rabbit sera R9 to Tecta used at 1:1000, R7 to Tectb, used at 1:500, and affinity purified rabbit anti-stereocilin antibody B2 used at 1:150, a gift from C.Petit (Institut Pasteur, France).

### Fluorescence microscopy of cochlear wholemounts

Labyrinths were fixed in 3.7% formaldehyde in PBS buffer pH 7.2 for 4 h at room temperature followed by ∼12 h at 4°C, washed in PBS, decalcified in 0.5 M EDTA for 2 days at 4°C and then washed and held in PBS. Individual cochleae were dissected following a video guide produced by Charles Liberman (http://www.masseyeandear.org/research/ent/eaton-peabody/epl-histology-resources/video-tutorial-for-cochlear-dissection/, last accessed date on December 17, 2013) with minor modifications to enable the entire organ of Corti to be harvested from four pieces. Dissected pieces were pre-blocked in 10% horse serum in PBS containing 0.1% Triton X-100 for 1 h followed by overnight incubation in 1:200 anti-myosin VIIa (Proteus) and 1:200 Texas Red phalloidin (Invitrogen). Pieces were then washed in PBS, stained for 2 h with 1:500 Alexa-488 goat anti-rabbit Ig (Invitrogen) diluted in PBS/HS, washed in PBS and mounted in Vectashield mounting medium under a no. 0 thickness glass coverslip. Shims were not used apart from for the basal-most piece, which was shimmed to a thickness of 0.4 mm. Confocal images of wholemounts were collected using a ×20 objective lens and manually photomontaged in Photoshop CS5. IHCs were counted, starting from the apical pole, and OHC loss was measured by counting the number of missing hair cells as judged by phalloidin staining of hair bundles and myosin VIIa staining of hair-cell bodies. OHC loss was counted instead of numbers of OHCs present as in many cases the natural variability in hair-cell numbers would have masked the hair-cell loss. As IHC loss was negligible apart from at the extreme basal end, distance from the apical end was measured as a function of IHC number, and missing OHCs were binned in 100 IHC groups, starting at a point 20 IHCs on from the apical end. The region containing the first 20 IHCs was discounted as the irregular arrangement of OHCs made it impossible to discern how many were missing in this area. In the few cases where small regions (less than ∼10 IHC) of dissection damage prevented counting, numbers were extrapolated from neighbouring regions.

### Measurement of prestin transcript expression by RT-qPCR

For each mutant mouse line cochlear tissues from three wild-type and three heterozygous littermates at P28 were dissected away from the bony capsule in RNA Later (Life Technologies, UK). Total RNA was then prepared from both cochleae from individual animals using Trizol (Life Technologies, UK) with an additional chloroform extraction to remove residual phenol. Precipitated total RNA was recovered by centrifugation at 14 500*g* for 30 min at 4°C, redissolved in 10 µl of RNase free water and its concentration and purity measured at 260 and 280 nm using a Picodrop spectrophotometer (Alpha Biotech, UK). First strand cDNA was synthesised from 1 µg of total RNA for each animal in a final volume of 20 µl, using an Im-Prom II Reverse Transcription System (Promega, UK).

All qPCR primers were designed using Primer3plus, (31), optimal *T*_anneal_ = 60°C, length = 20 bp. All primer pairs spanned an intron. Standards for qPCR were amplified from wild-type mouse cochlea cDNA using Accuprime HF (Life Technologies) or Pfu polymerase (Agilent) with the primers listed in Supplementary Material, Table S1. PCR products were confirmed to be a single product of the correct size by agarose gel electrophoresis and purified from the PCR reaction using Nucleospin columns (Machery-Nagel). The concentrations of the purified standards were calculated from their A_260_ using their calculated extinction coefficients. Standards were serially 10-fold diluted in 10 µg/ml yeast tRNA from 5 × 10^6^ copies/µl to 5 copies/µl. Aliquots (2 µl) were used in the qPCR to give a calibration range of 10^7^–10^1^ copies of standard per reaction.

qPCR was performed on a Stratagene A3005 qPCR machine (Agilent). PCR reactions contained Brilliant III Ultra Fast SYBR green qPCR master mix (Agilent), specific primers at 0.5 µM (Supplementary Material, Table S2) and 2 µl cDNA or prepared standards in a total volume of 10 µl. Duplicate reactions were used for each cDNA sample. All runs included an initial incubation for 3 min at 95°C followed by 40 cycles of denaturing at 15 s at 95°C, annealing for 15 s at 60°C, and extending for 20 s at 72°C, followed by melting curve analysis. In pilot experiments the PCR products were separated by agarose gel electrophoresis to confirm that only a single band of the correct size was amplified for each primer pair. All RT-qPCR analyses were linear (*r*^2^ > 0.99) and had efficiencies >95% on standards between 10^7^ and 10^1^ copies and on 2-fold diluted cDNA. Absolute quantification was performed using the standard curves and the data for prestin and myosin VIIa were normalised in GENorm using the normalisation factor calculated from TBP, Ywhaz and Pla2gl2a, the three most stably expressed control genes (32). Prestin copy numbers were then normalised to myosin VIIa as a proxy for hair-cell number and prestin copy number from +/- animals expressed as a percentage of wild-type prestin copy number.

### Measurement of ABRs

Hearing function was evaluated by measuring the threshold of the ABR in response to click and tone burst stimuli as described previously (33) with modifications. Briefly, mice were anaesthetized by intraperitoneal administration of ketamine (Imalgene^®^ 500, Merial, 100 mg/kg) and xylazine (Rompum© 2%, Bayer Labs, 10 mg/kg), and maintained at 37°C with a heating blanket throughout the testing period to avoid hypothermia. Acoustic stimulation and auditory evoked potential amplification and recording were performed with TDT System 3™ workstation and the specific software SigGenRP™ and BioSigGenRP™ (Tucker Davis Technologies TDT, Alachua, FL, USA).

### Noise exposure

Mice were placed individually in a Perspex-walled chamber within a ventilated, sound-proof box. White noise within the 8–16 kHz frequency range was delivered through tweeters and levels were increased every 10 s in small increments from ambient (∼37 dB SPL) to 102 dB SPL. SPL on the floor of the Perspex chamber were measured with an Extech Instrument digital sound level meter. Behaviour was recorded with a webcam mounted on the ceiling of the sound-proofed booth using Debut Video Capture software. Following preliminary experiments, sound was terminated within 5 s of the onset of wild running behaviour. A total of 47 wild-type and 38 heterozygous animals (18 Tecta^C1619S/+^, 11 Tecta^L1820F,G1824D/+^ and 9 Tecta^C1837G/+^) were tested using this procedure.

## SUPPLEMENTARY MATERIAL

Supplementary Material is available at *HMG* Online.

## FUNDING

This work was supported by The Wellcome Trust (087377 to G.P.R.), Fondo Investigaciones Sanitarias (PI08/0045, PI11/1215 to M.A.M.P.), the Fundación Ramon Areces (CIVP16A1849 to M.A.M.P.) and the EU (FP6 Integrated Project EuroHear to G.P.R. and M.A.M.P.). Funding to pay the Open Access publication charges for this article was provided by The Wellcome Trust.

## Supplementary Material

Supplementary Data
