# Supplementary material for: Three deaf mice: mouse models for TECTA-based human hereditary deafness reveal domain-specific structural phenotypes in the tectorial membrane
Source: Hum Mol Genet. 2013 Dec 20;23(10):2551–68. doi: 10.1093/hmg/ddt646 (PMC3990158; doi:10.1093/hmg/ddt646)
Supplement: Supplementary Data [file supp_23_10_2551__index.html]

Three deaf mice: mouse models for TECTA-based human hereditary deafness reveal domain-specific structural phenotypes in the tectorial membrane — Three deaf mice: mouse models for TECTA-based human hereditary deafness reveal domain-specific structural phenotypes in the tectorial membrane — Supplementary Data 

# Three deaf mice: mouse models for TECTA-based human hereditary deafness reveal domain-specific structural phenotypes in the tectorial membrane

## Supplementary Data

Supplementary Data

**Files in this Data Supplement:**

- Supplementary Data - Doc file
- Supplementary Figure 1 - tif file
- Supplementary Figure 2 - tif file
- Supplementary Figure 3 - tif file
