# Supplementary material for: Three deaf mice: mouse models for TECTA-based human hereditary deafness reveal domain-specific structural phenotypes in the tectorial membrane
Source: Hum Mol Genet. 2013 Dec 20;23(10):2551–68. doi: 10.1093/hmg/ddt646 (PMC3990158; doi:10.1093/hmg/ddt646)
Supplement: Supplementary Data [file supp_ddt646_ddt646supp.doc]

**Supplementary Data**

**Legends to Supplemental Figures**

Supplemental Figure 1. Apical region of a P30 cochlea stained with phalloidin (red in **A**, panel **B**) and antibodies to myosin VIIa (green in **A**, panel **C**). Missing hair cells are indicated by arrowheads, adjacent intact hair cells are indicated by arrows. Note that the pattern of supporting cells is retained around each missing hair cell (compare supporting cell patterns adjacent to arrowheads and arrows), allowing the number of lost hair cells to be determined accurately. Scale bar = 20 μm.

Supplemental Figure 2. Micrographs of the upper level of the TM from the sulcal region, close to the limbal attachment zone, in wildtype (**A**), *Tecta*^C1619S/+^ (**B**), *Tecta*^L1820F, G1824D/+^ (**C**) and *Tecta*^C1837G/+^(**D**) adult mice. Collagen fibrils erupt from the surface of the TM in *Tecta*^L1820F, G1824D/+^ (**C**) and *Tecta*^C1837G/+^(**D**) mice. Bar = 1 μm.

Supplemental Figure 3. Limbal-region tectorial membrane from a P70 Tecta^C1619S/+^ mouse showing giant collagen fibres. Panel **B** shows a higher magnification of a region from panel **A**. Arrows in **B** point to typical TM collagen fibrils that are in close proximity to the giant fibre. Note that the distinctive striations (arrowheads in **B**) of the giant fibre have a periodicity of 65-70 nm. Bar in **A** = 2 μm, in **B** = 500 nm.

**Supplemental Table 1**

Primers used to amplify qPCR standards

| Target | Accession No | Primer Name | Primer Sequence | Product size (bp) |
| --- | --- | --- | --- | --- |
| Tbp | NM_013684 | qMmTBPSF1 | gggcttcccagctaagttct | 499 |
|  |  | qMmTBPSR1 | cagcacagagcaagcaactc |  |
|  |  |  |  |  |
| Ywhaz | NM_01253805 | qMmYwhazSF1 | agcaggcagagcgatatgat | 486 |
|  |  | qMmYwhazSR1 | cagagaagttgagggccaga |  |
|  |  |  |  |  |
| Hmbs | NM_013551 | qMmHmbsSF1 | caccacgggagacaagattc | 391 |
|  |  | qMmHmbsSR1 | gacgatggcactgaattcct |  |
|  |  |  |  |  |
| Pla2gl2a | NM_023196 | qMmPla2gl2aSF1 | cactgtttggcgttcatctg | 321 |
|  |  | qMmPla2gl2aSR1 | tctccagcagtcagggtctt |  |
|  |  |  |  |  |
| Myosin7a | NM_008663 | qMmMyo7aSF1 | cgcactcatctacagggtca | 589 |
|  |  | qMmMyo7aSR1 | gtccagcttccctgcctaga |  |
|  |  |  |  |  |
| Prestin | AF529192 | qMmPresSF1 | tggcatttctgcaggattta | 471 |
|  |  | qMmPresSR1 | aggtctgagaactgcatgaaca |  |
|  |  |  |  |  |

**Supplemental Table 2**

Primers used to quantify gene expression by qPCR

| Target | Accession No | Primer Name | Primer Sequence | Product size (bp) |
| --- | --- | --- | --- | --- |
| Tbp | NM_013684 | qMmTBPF1 | gacccaccagcagttcagtag | 216 |
|  |  | qMmTBPR1 | caggagaacatggcagacaa |  |
|  |  |  |  |  |
| Ywhaz | NM_01253805 | qMmYwhazF1 | ttgagcagaagacggaaggt | 247 |
|  |  | qMmYwhazR1 | tgctgtgactggtccacaat |  |
|  |  |  |  |  |
| Hmbs | NM_013551 | qMmHmbsF1 | aagtggacctggtcgttcac | 196 |
|  |  | qMmHmbsR1 | agctgagccactctcctcag |  |
|  |  |  |  |  |
| Pla2gl2a | NM_023196 | qMmPla2gl2aF1 | gcacgacagatgctacgaga | 168 |
|  |  | qMmPla2gl2aR1 | tggatgacgctgtcaaagag |  |
|  |  |  |  |  |
| Myosin7a | NM_008663 | qMmMyo7aF2 | cccttcaccaagatctccaa | 208 |
|  |  | qMmMyo7aR2 | gcgtctcctctgaggttcac |  |
|  |  |  |  |  |
| Prestin | AF529192 | qMmPresF7 | gtctcgaagccttgttcagg | 154 |
|  |  | qMmPresR7 | caggttgacgatcacaatgg |  |
|  |  |  |  |  |

In all cases the products of primer pairs span at least one intron and generate a product from genomic DNA that is too large to be amplified during the 20s extension used in the qPCR.

Abbreviations: TM, tectorial membrane; ABR, auditory brainstem response; ADNSL, autosomal dominant non-syndromic hearing loss; ZP, zona pellucida; ZA, zonadhesin-like; TIL, trypsin inhibitor-like; vWF D, von Willebrand factor D repeat;
