# Supplementary figures and images for: Three deaf mice: mouse models for TECTA-based human hereditary deafness reveal domain-specific structural phenotypes in the tectorial membrane
Source: Hum Mol Genet. 2013 Dec 20;23(10):2551–68. doi: 10.1093/hmg/ddt646 (PMC3990158; doi:10.1093/hmg/ddt646)

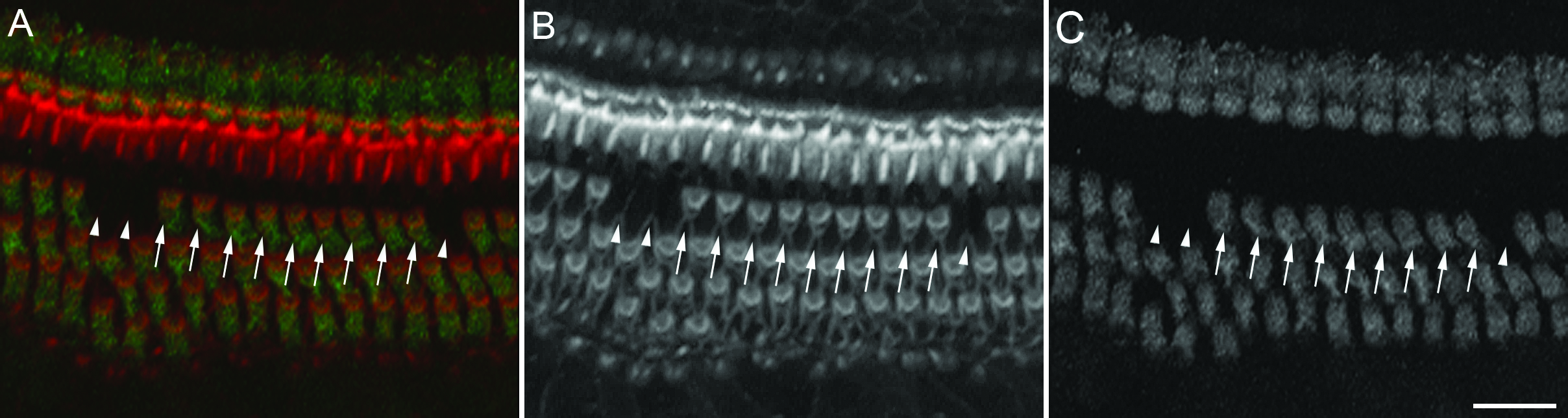

Supplement: Supplementary Data [file supp_ddt646_ddt646supp_fig1.tif]

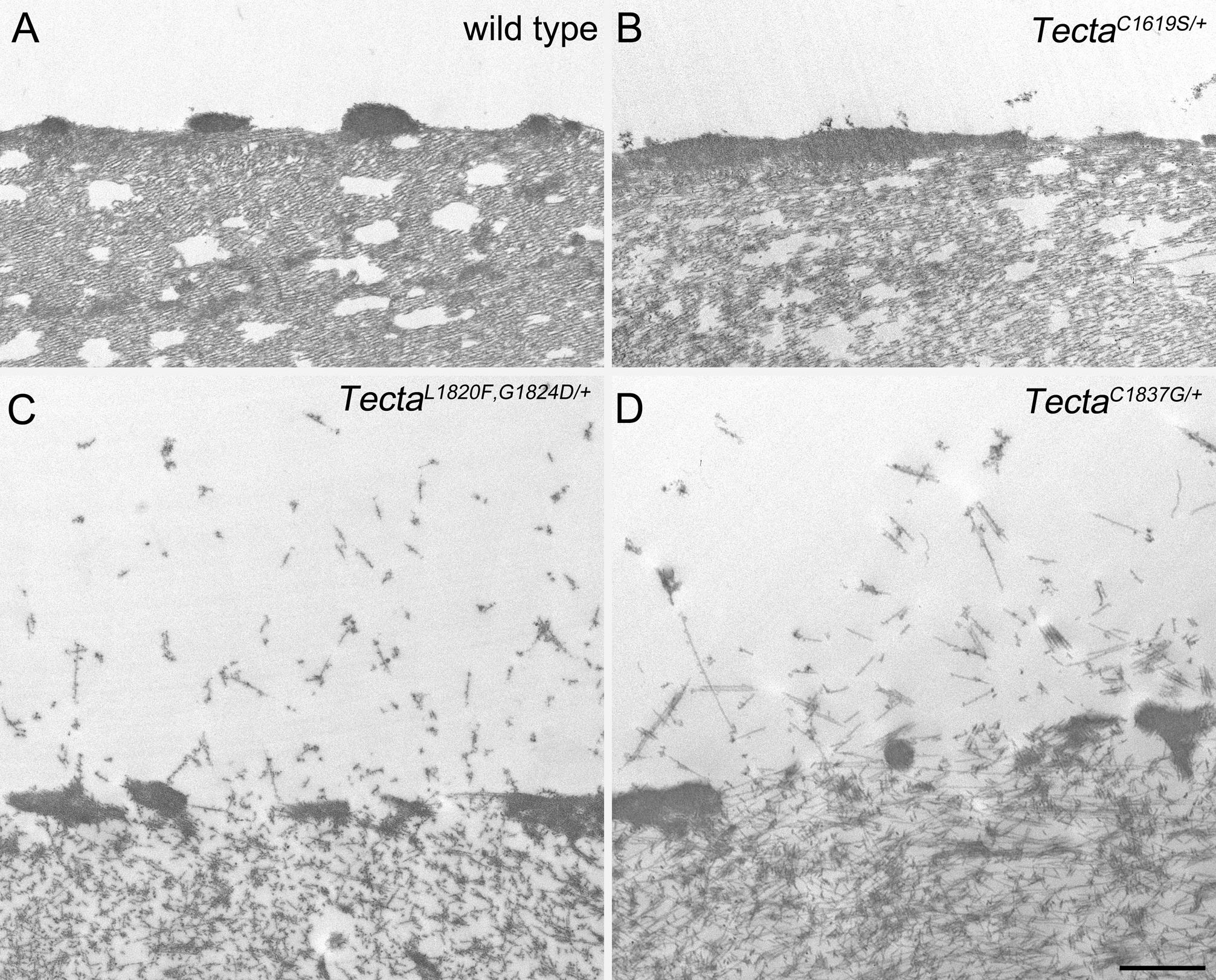

Supplement: Supplementary Data [file supp_ddt646_ddt646supp_fig2.tif]

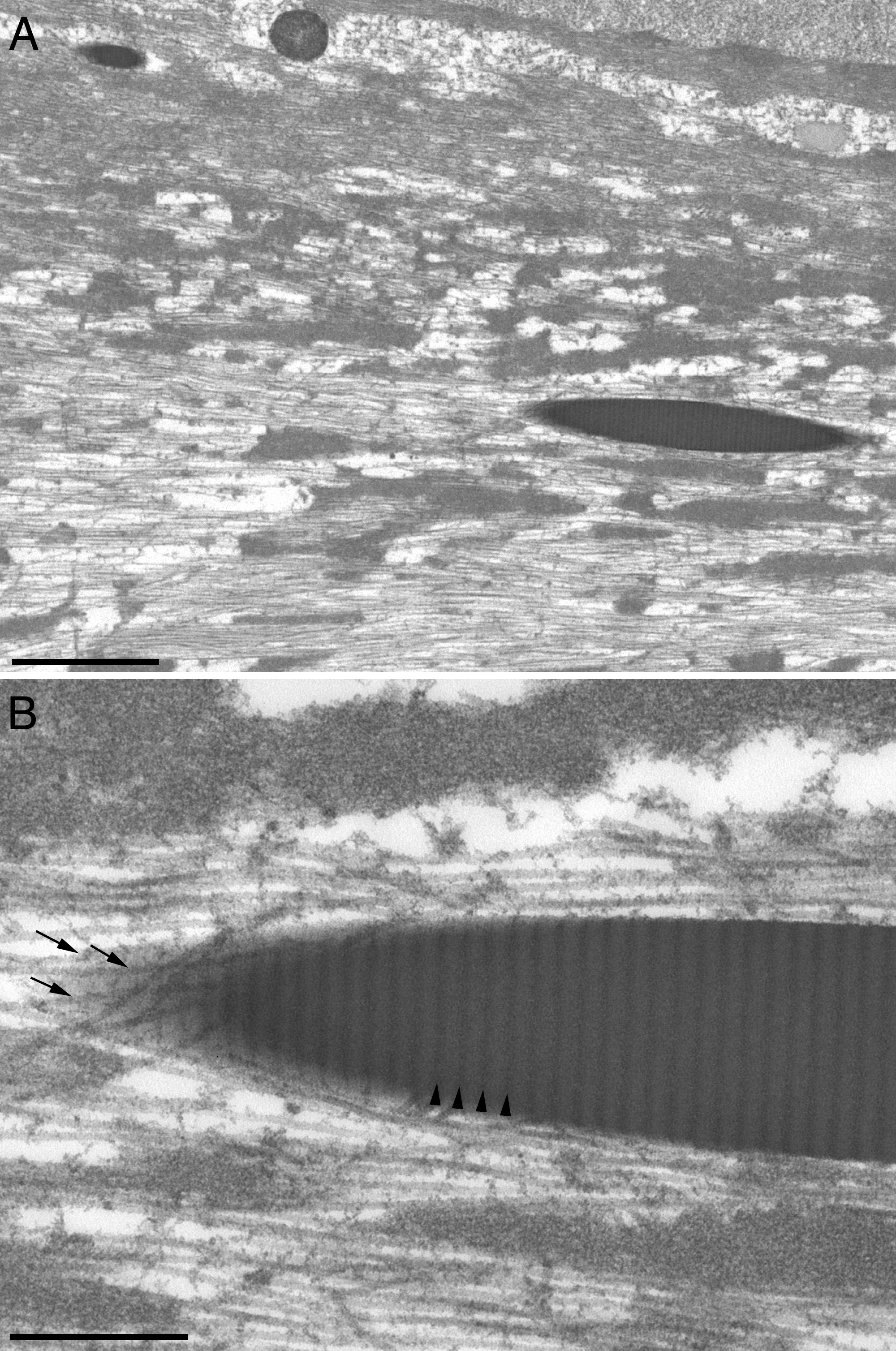

Supplement: Supplementary Data [file supp_ddt646_ddt646supp_fig3.tif]
